# Dysregulation of Calcium Handling in Duchenne Muscular Dystrophy-Associated Dilated Cardiomyopathy: Mechanisms and Experimental Therapeutic Strategies

**DOI:** 10.3390/jcm9020520

**Published:** 2020-02-14

**Authors:** Michelle L. Law, Houda Cohen, Ashley A. Martin, Addeli Bez Batti Angulski, Joseph M. Metzger

**Affiliations:** 1 Department of Family and Consumer Sciences, Robbins College of Health and Human Sciences, Baylor University, Waco, TX 76706, USA; Michelle_L_Law@baylor.edu; 2 Department of Integrative Biology and Physiology, University of Minnesota Medical School, Minneapolis, MN 55455, USA; cohen461@umn.edu (H.C.); aamartin@umn.edu (A.A.M.); abezbatt@umn.edu (A.B.B.A.)

**Keywords:** dilated cardiomyopathy, muscular dystrophy, calcium, heart, gene therapy, phospholamban, Serca2a, *mdx*, oxidative stress, membrane stabilization

## Abstract

Duchenne muscular dystrophy (DMD) is an X-linked recessive disease resulting in the loss of dystrophin, a key cytoskeletal protein in the dystrophin-glycoprotein complex. Dystrophin connects the extracellular matrix with the cytoskeleton and stabilizes the sarcolemma. Cardiomyopathy is prominent in adolescents and young adults with DMD, manifesting as dilated cardiomyopathy (DCM) in the later stages of disease. Sarcolemmal instability, leading to calcium mishandling and overload in the cardiac myocyte, is a key mechanistic contributor to muscle cell death, fibrosis, and diminished cardiac contractile function in DMD patients. Current therapies for DMD cardiomyopathy can slow disease progression, but they do not directly target aberrant calcium handling and calcium overload. Experimental therapeutic targets that address calcium mishandling and overload include membrane stabilization, inhibition of stretch-activated channels, ryanodine receptor stabilization, and augmentation of calcium cycling via modulation of the Serca2a/phospholamban (PLN) complex or cytosolic calcium buffering. This paper addresses what is known about the mechanistic basis of calcium mishandling in DCM, with a focus on DMD cardiomyopathy. Additionally, we discuss currently utilized therapies for DMD cardiomyopathy, and review experimental therapeutic strategies targeting the calcium handling defects in DCM and DMD cardiomyopathy.

## 1. Introduction

### 1.1. Dilated Cardiomyopathy: Prevalence, Causes, and Clinical Manifestations

Cardiovascular diseases (CVD) contribute to approximately 30% of global morbidity and mortality, representing a major public health concern [1]. Among the several types of CVD, dilated cardiomyopathies (DCM) are an important cause of congestive heart failure and cardiac disease requiring heart transplantation [2]. DCM is a disease of the heart muscle characterized by increased ventricle chamber volume and impaired systolic function involving the left or both ventricles. DCM can develop at any age, is a common form of cardiomyopathy in the pediatric population, and can lead to sudden cardiac death in adolescents and young adults [2,3]. Although the etiology of DCM remains unknown in 66% of cases, myocarditis and neuromuscular diseases are the most commonly recognized causes of DCM. In 20–48% of cases, the disease is inherited and is referred to as familial dilated cardiomyopathy [2,3].

DCM is commonly underdiagnosed because most individuals are asymptomatic in the early stages of the disease. Typically, DCM is diagnosed during screening for cardiac dysfunction in individuals considered at risk, such as in family members of DCM patients. Early symptoms are nonspecific and include easy fatigability, decreased appetite, effort-induced shortness of breath, intermittent chest pain, fainting, syncope, and/or palpitations [3]. Undiagnosed, patients will later present with symptoms of end organ dysfunction due to systolic defects and peripheral hypoperfusion [3]. Physical examination can reveal sinusoidal tachycardia, gallop rhythm, a heart murmur, jugular-venous distention, pallor, cool hands and feet, and hepatomegaly at more advanced stages. Patients with severe DCM demonstrate symptoms and complications of congestive heart failure, such as dyspnea with exertion or at rest, chest pain, abdominal pain, and peripheral edema. Arrhythmias, thromboembolism, and sudden death are also common in DCM and may occur at any stage [3,4].

### 1.2. Dilated Cardiomyopathy in Muscular Dystrophy: Prevalence, Clinical Manifestations

Duchenne muscular dystrophy (DMD) is the most common and a severe form of muscular dystrophy. It affects approximately 1 in 5000 males [5]. DMD is an X-linked myopathy caused by a mutation in the dystrophin gene resulting in a complete loss of dystrophin protein in striated muscles. Absence of dystrophin leads to disruption of the dystrophin-associated glycoprotein complex (DGC), which connects the cytoskeleton to the extracellular matrix and contributes to force transmission. Dystrophin is essential for stabilization of the sarcolemma. In DMD, loss of dystrophin leads to a fragile sarcolemma susceptible to stress-induced damage, resulting in myocyte death and progressive muscle wasting [6]. Early signs of muscle weakness in children affected with DMD generally occur between the ages of three and five. Muscle weakness initially involves the proximal muscles (Gowers’ sign), followed by distal skeletal muscle groups (limbs and trunk). DMD is progressive, and in the absence of appropriate treatment and care, affected individuals typically lose ambulation by age 10–12 [7].

Until more recently, early loss of mobility and respiratory complications in DMD patients obscured symptoms of cardiac involvement. Over the past two decades, clinical advancements in respiratory assistance [8,9], implementation of anti-inflammatory drugs, and emerging muscle-targeted therapies led to improvement of activity levels, extended ambulation, and increased longevity in the DMD population. This, in turn, revealed cardiomyopathy as a major cause of morbidity and mortality in DMD patients [10,11,12]. It is estimated that 25% of boys have cardiomyopathy at 6 years of age and 59% by 10 years of age [13]. Cardiac involvement becomes highly prominent as DMD boys advance in age, with more than 90% of young men over 18 years of age having evidence of significant cardiac dysfunction [13].

The development of dilated cardiomyopathy in DMD is a consequence of multiple mechanisms, and a complete understanding of the pathophysiology has not been elucidated. The elevation of plasma creatine kinase (CK) is a hallmark of DMD, indicating that there is increased permeability of the plasma membrane, allowing soluble enzymes to leak out of the muscle cell [14,15,16]. The absence of dystrophin disrupts force transmission and causes contraction-induced sarcolemma damage and membrane permeability that allows an influx of calcium, triggering death of the myocyte [14,15,16]. The mechanisms that lead to increased permeability of the plasma membrane are not fully understood; however, it is widely acknowledged that increased calcium influx and calcium overload contribute to the molecular progression of the disease [17,18,19,20]. In the earlier stages of disease, compensatory mechanisms have been noted. In particular, an increase in calcium transient amplitude, increased sarcoplasmic reticulum calcium load and leak, and a reduced cardiac reserve are reported in animal models [21,22]. In the later decompensated stage, DMD cardiomyopathy presents as DCM, characterized by enlarged ventricles, reduced systolic function, decreased calcium transients, reduced cardiac wall thickness, and cardiac arrhythmias [3,23].

### 1.3. Objective of This Review

Understanding in greater detail the underlying mechanisms of DCM pathogenesis in DMD is critical to the development of targeted therapies for this disease. Current advancements in the treatment of skeletal muscle pathology may not benefit dystrophic cardiac muscle in the same way due to key differences in the function and calcium handling of these two striated muscle lineages [24]. Further, treatment of skeletal muscle without regard to the heart may exacerbate cardiac dysfunction [25], as can be seen in patients with X-linked dilated cardiomyopathy. These patients exhibit normal dystrophin expression in skeletal muscle and the absence of dystrophin in the heart [26,27]. Indeed, improved treatment options focused on ameliorating the skeletal muscle pathology of DMD have uncovered a previously underappreciated cardiac involvement, with an estimated 20–30% of deaths now resulting from cardiac failure in this population [28]. The purpose of this paper is to (1) discuss the role of calcium mishandling in DCM development and progression with a focus on DMD cardiomyopathy, (2) detail currently utilized therapies for DMD cardiomyopathy, and (3) evaluate experimental therapeutic strategies for correcting calcium mishandling and calcium overload in DCM and DMD cardiomyopathy.

## 2. Molecular Mechanisms of Dilated Cardiomyopathy

### 2.1. Genetic and Acquired Causes of DCM

**Figure 1:**
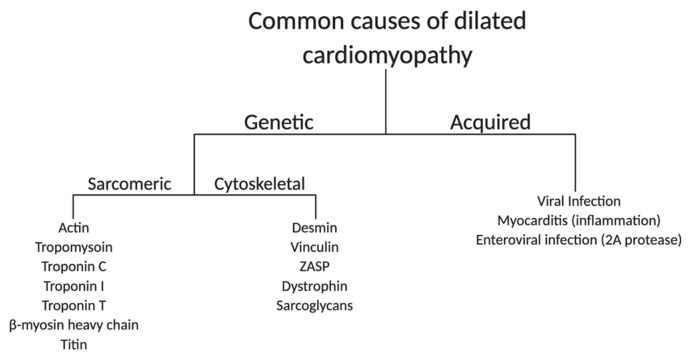
Common genetic and acquired causes of dilated cardiomyopathy in humans. ZASP: z-band alternatively spliced PDZ-motif.

There are a multitude of genetic and acquired causes of DCM, making the pathology of the disease highly diverse and clinically vexing [23]. Many known genetic and novel mutations leading to DCM are contained within the sarcomere or cytoskeleton. Therefore, these structures will be the main focus here. A schematic outlining common causes of DCM is shown in Figure 1.

#### 2.1.1. Sarcomere

The cardiac sarcomere consists of a repeating pattern of contractile and regulatory proteins organized into thick and thin myofilaments [29]. The sarcomere is the basic contractile unit of cardiac muscle. Therefore, mutations impacting sarcomeric protein function can have a severe effect on heart performance. Approximately 5–10% of DCM diagnoses are due to mutations in sarcomeric proteins [30]. Thin filament proteins, including actin, tropomyosin (Tm), and the three protein subunits that make up the troponin complex, are common targets of DCM-causing mutations [31]. Mutations in the troponin complex typically cause a reduction in myofilament calcium sensitivity and force production [32]. There is evidence however, that in end stage heart failure, there is an increase in myofilament calcium sensitivity [33,34]. This increase is thought to be due to changes in phosphorylation levels of myofilament proteins such as troponin I (TnI), possibly through protein kinase A (PKA) [35,36,37]. Another site of DCM mutations is within thick filament proteins, including the MYH7 gene (variant pAsn1918Lys), which encodes β-myosin heavy chain, the primary mechano-motor protein of the adult human heart [38]. These mutations are thought to disrupt the ability of myosin to interact with actin, leading to a contractility defect [30]. Finally, a sarcomeric protein that has recently been identified as a target of DCM mutations is the giant protein titin [39]. Titin is a multifaceted protein that plays a key role in sarcomere assembly and helps maintain passive tension in muscles [40]. Mutations leading to the truncation of the titin protein account for 1–3% of DCM diagnoses, with disease characterized by significant diastolic dysfunction, highlighting the complex role of titin in regulating contraction of the sarcomere [23].

#### 2.1.2. Cytoskeleton

Mutations in genes encoding cytoskeletal proteins in cardiac muscle are also highly associated with DCM. The loss of dystrophin results in DCM [41], including X-linked DCM where heart dysfunction and failure can be highly pronounced [27]. Mutations in proteins that interact with dystrophin, such as the sarcoglycans, can also represent causal genetic loci for DCM [42]. Although these proteins are found in both skeletal and cardiac muscle, mutations in some of these proteins can cause heart disease without significant skeletal muscle deficits [43]. Other commonly targeted proteins include desmin, vinculin, and z-band alternatively spliced PDZ-motif (ZASP) [44,45]. These proteins are integral for maintaining the connection between muscle fibers, the sarcolemma, and the extracelluar matrix. Mutations in cytoskeletal proteins generally inhibit myocyte force transmission and increase muscle membrane instability and permeability. The compromised sarcolemma leaves the muscle cell susceptible to increases in intracellular calcium, which can result in calcium overload and myocyte death [41,46].

#### 2.1.3. Acquired Causes of DCM

DCM can also be acquired through mechanisms unrelated to genetic factors. Causes of DCM outside of familial inheritance include infection or ischemia, which can lead to myocarditis [47]. Viral infection can cause both damaging immune and inflammatory responses and direct viral toxicity, resulting in cardiac myocyte necrosis, fibrotic development, and ventricular dilation [48]. Enterovirus infection can also cause cardiomyopathy, and in its most severe form, sudden death. The mechanism of disease is thought to be through the virus-encoded 2A protease, which cleaves the dystrophin protein. The resulting dystrophin fragment increases risk for fibrosis and ischemic injury *in vivo* [49]. Reductions in myocardial perfusion, a known deficit caused by ischemic heart disease, is also observed in DCM patients [50]. Defects in myocardial blood flow can lead to chronic ischemic events and thus contribute to the progression of DCM [51].

### 2.2. The Role of Calcium Cycling in DCM Pathogenesis

#### 2.2.1. Calcium Cycling in Healthy Cardiac Myocytes

**Figure 2:**
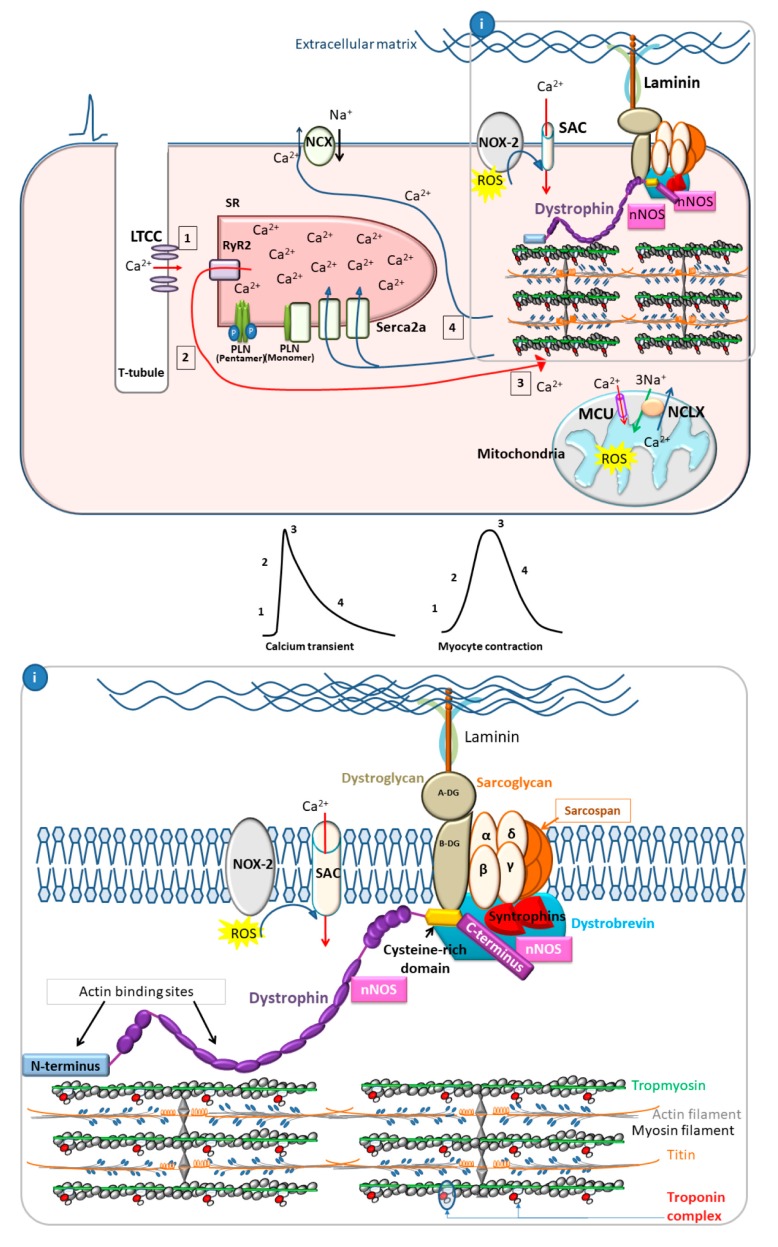
Normal excitation-contraction coupling in cardiac myocytes. Membrane depolarization leads to a small influx of calcium through the L-type calcium channel (LTCC/DHPR) (1), which triggers a larger release of calcium from the sarcoplasmic reticulum (SR) through ryanodine receptor 2 (RyR2) (2). Calcium then binds to the myofilaments, triggering myocyte contraction (3). During the relaxation phase, calcium reuptake occurs by pumping calcium out of the cytoplasm back into the SR via Serca2a or through the Na^+^/Ca^2+^ exchanger (NCX) (4). Phospholamban negatively regulates Serca2a activity. β-adrenergic signaling leads to phospholamban (PLN) phosphorylation and dissociation from Serca2a, increasing the rate of calcium reuptake into the SR. Normal physiological stretch leads to NADPH oxidase 2 (NOX-2) production of reactive oxygen species (ROS), which increases calcium entry through stretch-activated channels (SACs). Dystrophin serves to stabilize the sarcolemma during the repeated stress of myocyte contraction and relaxation. Inset shows increased detail of the dystrophin glycoprotein complex (DCG) and myofilament proteins. NOS: nitric oxide synthase; MCU: mitochondrial calcium uniporter; NCLX: mitochondrial sodium calcium exchanger.

During contraction in healthy cardiac myocytes, electrical stimulation of the muscle leads to an increase in intracellular calcium, first as a small amount which enters through L-type voltage-gated calcium channels (dihydropyridine receptors (DHPR)) in the sarcolemma. The initial calcium influx then triggers a larger calcium release from the sarcoplasmic reticulum (SR) through ryanodine receptor 2 (RyR2), located in the SR membrane. Together, this process is referred to as calcium-induced calcium release (CICR) [52]. Calcium rises in the cytoplasm and binds to troponin C (TnC), causing the protein to undergo a conformational change, which is facilitated by the binding of troponin I (TnI). As TnI switches binding from actin to TnC, tropomyosin (Tm) is then free to move, exposing myosin-binding sites on the actin filaments [29]. Troponin T (TnT)-Tm binding allows for the cooperative transmission of these conformational changes along the length of the thin filament in a complex series of protein-protein interactions, and cross-bridge binding of myosin to actin stabilizes Tm positioning [29]. Through these interactions the myofilament becomes activated and force can be generated [52] (Figure 2).

During relaxation, SR-associated proteins, notably sarco(endo)plasmic reticulum Ca^2+^-ATPase (SERCA2a) and phospholamban (PLN), are essential to the removal of calcium from the cytoplasm back into the SR [53]. The Serca2a/PLN complex is the major regulator of calcium cycling in cardiac muscle [54]. Serca2a is an ATP-dependent calcium pump localized within the longitudinal membrane of the sarcoplasmic reticulum [55]. Serca2a has a key role in regulating both the rate of calcium reuptake and subsequent myocyte relaxation in diastole, as well as SR calcium load, which affects calcium transient peak height and contractility in systole [54,56]. PLN is a negative regulator of Serca2a function [54]. The monomeric, dephosphorylated form of PLN interacts with Serca2a and decreases its affinity for calcium, thus decreasing SR calcium reuptake velocity. Phosphorylation of PLN at Ser 16 or Thr 17 inhibits its interaction with Serca2a and increases oligomerization of PLN into its pentameric form, thereby relieving Serca2a inhibition [54,56]. The resulting increase in Serca2a function increases the rate of calcium reuptake in diastole and also increases SR calcium load, resulting in increased contraction amplitude and hastened relaxation (Figure 2).

The phosphorylation status of PLN is what determines its ability to alter Serca2a function. PLN phosphorylation is regulated in large part through β-adrenergic signaling. Stimulation of the sympathetic nervous system leads to increased activation and signaling through β-adrenergic receptors. The subsequent Protein Kinase A (PKA) activation leads to phosphorylation of PLN at Ser 16, relieving its inhibition on Serca2a and increasing SR calcium reuptake [54,56]. Phosphorylation at Thr 17 also relieves inhibition on Serca2a and is accomplished via Ca^2+^/CaM Kinase in response to physiological stressors including ischemic injury, pacing stress or increased calcium concentration [54,56]. Multiple accessory proteins, including HS-1 associated protein X-1 (HAX-1), Protein-Phosphatase 1 (PP1), endogenous inhibitors of PP1 (I-1 and I-2), Heat Shock Protein 20 (HSP20), and S100A1 proteins, are also associated with the Serca2a/PLN complex and serve to modulate PLN inhibition of Serca2a under various physiological conditions [54].

#### 2.2.2. Calcium Cycling Defects in DCM

**Figure 3:**
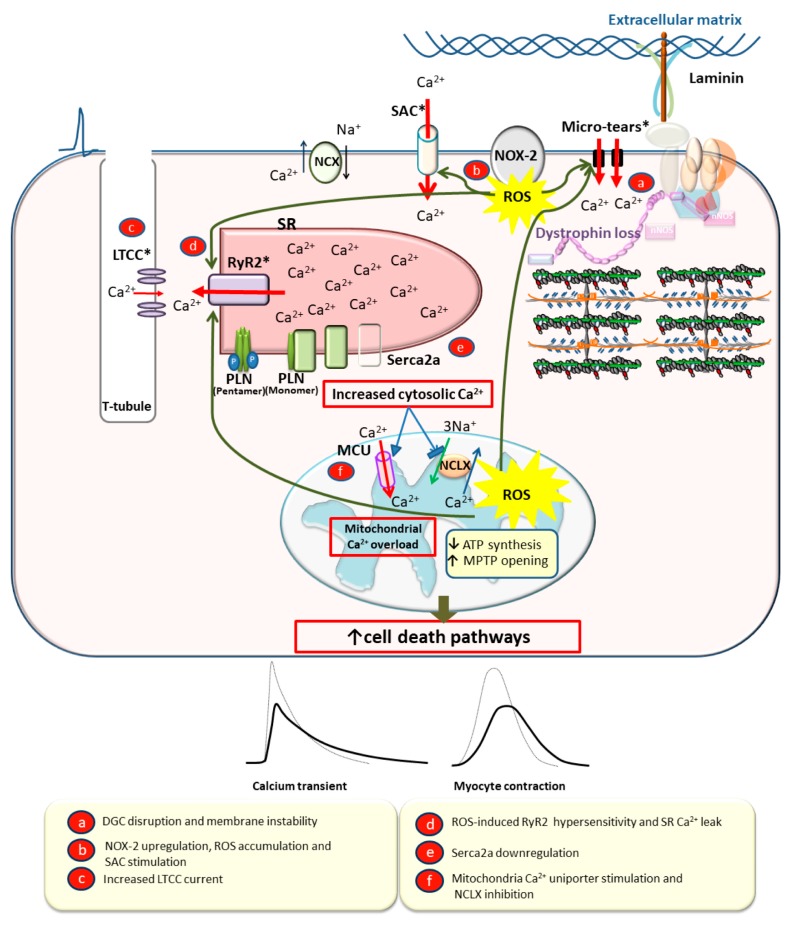
Mechanisms of calcium overload in dystrophin-deficient cardiac myocytes. The absence of dystrophin destabilizes the sarcolemma and leads to stress-induced membrane damage/micro-tears and calcium influx (**a**). Excessive reactive oxygen species (ROS) production in cardiac myocytes leads to further membrane damage and increased calcium influx via stretch-activated channels (SACs) and ryanodine receptor 2 (RyR2) (**b**). Increased L-type calcium channel (LTCC/DHPR) current also contributes to increased intracellular calcium (**c**). Calcium leak from RyR2 (**d**), decreased Serca2a expression (**e**) and increased phospholamban (PLN) inhibition of Serca2a decrease sarcoplasmic reticulum (SR) calcium load, subsequently decreasing calcium transient peak height and decay rate and inhibiting contractile function in later stages of Duchenne muscular dystrophy (DMD) cardiomyopathy (dark lines in transients). Increased cytosolic calcium leads to mitochondrial cell death pathways (**f**). * Indicates points of abnormal calcium entry into the myocyte. NCX: Sodium calcium exchanger; NOX-2: NADPH oxidase 2; NOS: Nitric oxide synthase; MCU: Mitochondrial calcium uniporter; NCLX: Mitochondrial sodium calcium exchanger.

DCM-causing mutations affect multiple aspects of calcium handling within the myocyte, from altering calcium sensitivity of the myofilament seen with sarcomeric mutations, to mislocalization and altered expression or function of calcium handling proteins seen with cytoskeletal mutations. In DCM, there is a decrease in peak height of the calcium transient in systole and a decreased rate of calcium reuptake in diastole [57]. Increased calcium leak from RyR2 can be a contributing factor, particularly in DMD cardiomyopathy [58]. The resulting decrease in SR calcium load decreases contractile function in systole [59]. Decreased calcium cycling and decreased SR calcium load can also occur via a decrease in the expression and/or activity of Serca2a [59,60,61]. PLN expression is not decreased to the same extent in DCM, thus increasing the ratio of PLN to Serca2a [60,61], which results in increased PLN inhibition of Serca2a. Additionally, β-adrenergic desensitization results in reduced PLN phosphorylation, which further increases its inhibition of Serca2a [54,60] (Figure 3).

## 3. Molecular Mechanisms of DMD Cardiomyopathy

The lack of dystrophin in cardiac myocytes leads to instability of the sarcolemma, resulting in calcium overload and oxidative stress. Over time, excess calcium and reactive oxygen species (ROS) activate cell death pathways, fibrosis, and dilation [12]. How dystrophin functions to stabilize the cell membrane is not fully understood, but two prevailing ideas are supported by the literature. First, dystrophin, as part of the DGC, connects the extracellular matrix and the intracellular cytoskeleton and acts as a “shock absorber” for the sarcolemma during the repeated stress of contraction and relaxation [62,63,64]. Sarcolemmal stress in the absence of dystrophin leads to muscle membrane damage or micro-tears, causing extracellular calcium influx and calcium overload [20,62,63] (Figure 3). Secondly, dystrophin acts as a scaffolding protein to localize and normalize function of proteins involved in intracellular calcium and redox homeostasis. Lack of dystrophin can cause mislocalization and abnormal expression/activity of these proteins, leading to calcium mishandling and oxidative stress [65]. Oxidative stress damages the sarcolemma and increases intracellular calcium entry through stretch-activated channels (SACs) in the sarcolemma [20] and RyR2 in the SR [65,66]. (Figure 3).

### 3.1. Dystrophin as a Membrane Stabilizer

When dystrophin is absent, as in DMD, the sarcolemma of cardiac myocytes is less compliant, which is revealed during passive length distension [41]. This leads to increased sarcolemma damage evidenced by lactate dehydrogenase (LDH) release under normal preload and afterload conditions in isolated working hearts [67]. This damage is even more pronounced under stress with isoproterenol or partial aortic constriction [67]. Damage is thought to occur via small membrane disruptions (micro-tears) that can lead to transient extracellular calcium influx [62] (Figure 3). Extracellular calcium influx raises intracellular calcium concentration, subsequently activating calcium release from the SR and increasing calcium concentration even further. Calcium overload ultimately results in myocyte hypercontracture and cell death [41,68].

Evidence for membrane destabilization as a primary cause of calcium overload and cell death in DMD comes from studies employing membrane stabilizers in the context of dystrophin deficiency. Membrane stabilizers, most notably the tri-block copolymer P188, have shown efficacy in reducing stress-induced calcium overload, hypercontracture, and cell death in animal models of DMD [41,68]. Membrane stabilizer studies provide evidence that dystrophin confers protection against stress-induced mechanical damage to the sarcolemma, and stabilizing damaged portions of membranes can prevent calcium overload and myocyte death.

### 3.2. Dystrophin as a Scaffold Protein

In addition to its function as a membrane stabilizer, accumulating evidence suggests a role for dystrophin in regulating ROS production and calcium handling within the myocyte. Dystrophin serves as a scaffold, helping to localize multiple proteins involved in calcium and oxidative homeostasis within the cell [65]. This allows for spatiotemporal control of ROS production and downstream signaling [66]. ROS products are important signaling molecules within the myocyte that regulate calcium cycling during physiological changes in cardiac load [69] (Figure 2). Abnormal or excessive ROS signaling in the absence of dystrophin [70] may contribute to cardiac pathology through aberrant calcium handling [70] (Figure 3).

Increased ROS production in DMD contributes to calcium overload from intracellular sources, namely the SR [66]. Indeed, ROS was found to underlie the hypersensitivity of RyR2 to increasing intracellular calcium concentrations in *mdx* cardiac myocytes, a mouse model of DMD [71]. Physiological stretch of isolated cardiac myocytes was found to induce a localized, rapid and transient increase in RyR2 calcium spark production, and this effect was amplified in *mdx* cardiac myocytes, leading to calcium waves [72]. Antioxidant treatment, microtubule depolymerization, and inhibition of reduced nicotinamide adenine dinucleotide phosphate (NADPH) oxidase 2 (NOX2) all abrogated this effect. It was concluded from this study that myocyte stretch caused microtubule activation of NOX-2, which increased NOX-2 ROS production, sensitized RyR2 and subsequently increased calcium spark frequency [72]. This is a physiologically important mechanism to increase calcium release during increased cardiac load. However, in the context of the dystrophin-deficient myocyte, this effect is amplified [72] and may be a contributing factor to increased diastolic calcium concentration [18] (Figure 3). *Mdx* mice have increased expression and density of microtubules [73], increased expression and activity of NOX-2 [72], and a compromised endogenous reducing system [66], all of which may lead to enhanced production of ROS and increased RyR2 calcium leak. Taken together, these studies reveal an important role for dystrophin in proper function and activity of proteins involved in both ROS and calcium handling within the myocyte.

### 3.3. Calcium Overload Leading to Myocyte Death, Fibrosis, and Dilation

Increased intracellular calcium in the context of dystrophin deficiency is a key mediator in myocyte death and fibrotic development [12]. As discussed above, excess calcium originates from both intracellular stores through hypersensitivity of RyR2 and from extracellular influx via sarcolemmal micro-tears and increased SAC activity [20]. Additionally, there is evidence for increased L-type calcium channel (LTCC) activity, which increases calcium cycling to compensate for myocyte loss and decreased β-adrenergic activity in young *mdx* mice [22]. This is an additional avenue for intracellular calcium overload and a potential cause of arrhythmias in DMD [74]. Increased calcium concentration in the cytosol leads to activation of calcium-dependent proteases and protein degradation [12,75] (Figure 3).

Increased intracellular calcium also causes an increase in mitochondrial uptake of calcium. Increased mitochondrial calcium concentration occurs via the mitochondrial uniporter (MCU) as a consequence of RyR2 calcium leak [76] or increased LTCC calcium current [77]. Additionally, high cytosolic calcium can inhibit mitochondrial calcium extrusion via the mitochondrial sodium-calcium exchanger (NCLX) [78]. Increased mitochondrial calcium leads to enhanced mitochondrial ROS production, depolarization of the mitochondrial membrane, opening of the mitochondrial permeability transition pore (MPTP) [79,80] and decreased ATP production. Additionally, it was found that impaired communication between the LTCC and mitochondria in the absence of dystrophin decreases mitochondrial membrane potential and energy production [81]. The end result of these processes is cell death via necrosis and apoptosis [12,75,79,80] (Figure 3). Mitochondrial-mediated cell death was found to be an important contributor to disease progression and fibrosis development in multiple animal models of muscular dystrophy [82]. Deletion or chemical inhibition of cyclophilin-D, an enzyme that regulates mitochondrial-mediated necrosis resulting from excess calcium [83], improved the dystrophic phenotype and decreased the replacement of healthy myocytes with fibrotic tissue [82].

Myocyte death, as a result of calcium overload, leads to the release of intracellular components and enzymes that initiate an inflammatory response. Clinically, DMD myocardium displays alternating areas of myocyte hypertrophy, atrophy/necrosis and fibrosis with replacement of heart muscle by connective tissue and fat [84,85,86]. DCM progression in DMD is characterized by a distinctive pattern of fibrosis, initially affecting the posterobasal myocardium of the left ventricular free wall, progressing to the ventricular septum, and extending transmurally to affect the outer half of the ventricular wall [87]. There is likely a long subclinical phase of progressive fibrosis that starts early in the course of the disease [88,89,90]. The development of progressive fibrosis will eventually lead to overt cardiac disease, dilation, and decreased pump function.

## 4. Model Systems to Study DCM and DMD Cardiomyopathy

To clarify the mechanisms responsible for the physiologic features of DCM and DMD cardiomyopathy, and to establish novel, preventative, and innovative therapies, several experimental animal and cell models of DCM and DMD cardiomyopathy have been developed and investigated. Most models recapitulate several clinical features of DCM in humans, typically exhibiting dilation of the left or both ventricles, severe impairment of systolic and diastolic left ventricle function, and thinning of the left ventricle wall [91]. This section will highlight both animal and cell models that have been developed to study DCM as an independent disease and models that study it as a pathology of DMD. An overview of this section is summarized in Table 1.

### 4.1. Rodents

Rodent models are commonly used in cardiovascular research as they are easier to handle and house (leading to manageable costs), and have a relatively short life span, allowing the researcher to follow the natural history of the disease. Additionally, of great impact is the capacity to leverage mouse genetic manipulation for both gain/loss of function of specific genes. This includes capacity for temporal control of tissue-specific genetic constructs [115].

To evaluate the development, progression, and potential for regression of DCM, multiple genetically engineered rodent models have been developed. These models include constitutive and inducible transgenic overexpression and/or gene knockout that exhibit a DCM phenotype [116,117]. One of the first DCM mouse models to be described was the muscle Lin11, Isi1 & Mec-3 (LIM) protein (MLP)-null mouse. Deletion of MLP, an actin-associated cytoskeletal protein, leads to cardiac myocyte architectural disorganization through irregularities in the actin-based cytoskeletal structure. Mice deficient for MLP show many of the anatomical and physiological hallmarks of human DCM [92]. Desmin-deficient models are also commonly used, which exhibit severe loss of myocardial architecture by degeneration and calcification [93]. Additionally, models with mutations in mitochondria can develop DCM with atrioventricular block due to deficient oxidative phosphorylation [91].

Several non-genetic methods, including drug and surgical techniques, are also used to induce the development of DCM in rodents. Surgical techniques include interruption of coronary arteries to produce myocardial infarction through permanent coronary ligation [94] or re-perfused infarction [95]. After an infarction, the DCM phenotype progressively develops in mice. Chronic doxorubicin [96,97] or isoproterenol [98,99] administration can lead to a dose-dependent dilated phenotype and overt heart failure over time owing to severe myocardial injury and cell death. Toxic drug-mediated cardiomyopathy is characterized by myocyte apoptosis and oxidative stress being highly specific forms of injury, which may also be useful in assessing cardiac responses to stress. It should be noted, however, that although these non-genetic models recapitulate many aspects of a DCM phenotype, DMD-associated DCM has noted differences in pathophysiology compared to these models.

*Mdx* mice that lack dystrophin are the most commonly used mouse model to study DMD. However, compared to patients with DMD, they exhibit a relatively minor cardiac phenotype [100,101]. Under baseline conditions, *mdx* mice do not demonstrate physiological indicators of heart failure early in life. However, disease can be readily unmasked by cardiac stressors. In efforts to make the baseline *mdx* cardiac phenotype more similar to that of patients, *mdx* mice have been crossed with utrophin knockout (KO) mice, which exhibit a more severe cardiomyopathy [102,103] and display the physiological indicators of end-stage heart failure, including a negative force-frequency relationship and a reduction in force development and impairment of relaxation [101]. Other symptomatic double knock-out strains have been generated by mutating genes involved in: (1) the DGC complex, including δ- sarcoglycan and dystrobrevin [118,119]; (2) muscle repair, such as dysferlin [120,121]; and (3) cytoskeleton-ECM interactions, including desmin and laminin [122,123]. A thorough characterization of the cardiomyopathy in these models will increase the usefulness of these animal models for research into treatments and diagnostics for DMD cardiomyopathy.

### 4.2. Large Mammals

Although rodent animal models are commonly used in cardiovascular research and may display some of the characteristics of human cardiac disease, they typically do not recapitulate all aspects of DCM found in humans [124,125]. Preclinical validation of therapeutic approaches can be advanced in large animal models due to their proximity to human cardiac physiology and structure [126]. Canines, swine, sheep, and non-human primates have been the most frequently used large animal models for cardiovascular research [104]. Taking into account the similarities in coronary anatomy, organ size, immunology, and physiology compared to humans, swine are considered the most attractive model for pre-clinical studies [105]. DCM can be induced in large animals by myocardial infarction, coronary micro-embolization, pacing-induced tachycardia, and toxic injury. Infarction models, including both re-perfused and non-re-perfused approaches in dogs [106], pigs [107], and sheep [108], are used to evaluate the pathophysiological mechanisms of post-infarction remodeling as well as DCM development, progression, and therapeutic response.

The availability of large animal models of DMD has been instrumental in gaining insights into the cardiomyopathy and progression to heart failure associated with DMD. The golden retriever muscular dystrophy (GRMD) canine model of DMD has been indispensable, not only for the development of therapeutic approaches, but also for the study of the pathobiology of dystrophin deficiency in the heart [68,109,110]. Muscular dystrophy in GRMD animals closely recapitulates the timing and severity of disease progression observed in DMD patients. In addition to the severe skeletal muscle pathology, GRMD animals have prominent cardiac lesions present as early as 6 months of age [127], with ECG abnormalities present at 1 year [128] and profound myocardial contractile abnormalities by 20 months [129].

### 4.3. Human iPSCs

The contribution of animal models to our overall understanding of DCM has been indispensable. However, many important differences exist between animal models and humans. Additionally, cardiac tissues from DCM patients are difficult to obtain and exhibit a low survival rate in long-term culture. The emergence of induced pluripotent stem cells (iPSCs) [130], and the rapidly advancing technology associated with them have made it possible to obtain functional cardiac myocytes through the differentiation of human iPSCs derived from DCM patients [113,114]. Stem cell-derived cardiac myocytes from cells isolated directly from patients with cardiomyopathies recapitulate certain aspects of human cardiovascular disease and represent a powerful new model system to study the basic mechanisms of inherited cardiomyopathies. Thus, human induced pluripotent stem cell-derived cardiac myocytes (hiPSC-CMs) are an important complement to experimental animal models to study the cellular, molecular, and physiological mechanisms associated with the pathogenesis of DCM, as well as to establish high-throughput platforms for drug screening in human cells.

DCM was first modeled by Sun and coworkers using iPSCs-CMs derived from a member of a family with DCM carrying a heterozygous R173W mutation in cardiac troponin T (TNNT2) [111]. iPSC-derived cardiac myocytes from this patient recapitulated some of the morphological and functional phenotypes of familial DCM with inherited mutations in troponin T. This study describes the first successful modeling of dilated cardiomyopathy in hiPSC-CMs. Another patient-specific DCM iPSC line was generated from a single member of a family with an autosomal dominant nonsense mutation (p.R225X) in exon 4 of the lamin A/C (LMNA) gene. hiPSC-CMs from this patient showed morphologic changes, including a higher prevalence of nuclear bleb formation, micronucleation, as well as nuclear senescence and cellular apoptosis [112]. Additionally, Tse and colleagues generated hiPSC-CMs derived from a DCM patient with a novel heterozygous mutation of p.A285V codon conversion on exon 4 of the desmin (DES) gene [113]. In this study, hiPSC-CMs were able to provide histologic and functional confirmation that the candidate gene variant detected by whole exome sequencing was responsible for the disease.

To study the molecular mechanisms underlying DCM in DMD, Lin and co-workers generated cardiac myocytes (CMs) from DMD patients and healthy control induced pluripotent stem cells (iPSCs). Using DMD patient-derived iPSC-CMs, they have established an *in vitro* model that manifests the major phenotypes of DCM in DMD patients, and uncovered a potential new disease mechanism [114]. In this regard, Lin and co-workers examined a collection of muscular dystrophies (including DMD and Becker Muscular Dystrophy) and healthy hiPSC-derived cardiac myocytes. This included demonstration that loading of the treated DMD hiPSC-derived cardiac myocytes with the calcium sensitive dye, Rhod-2AM, revealed increased cytosolic calcium concentration. The use of calcium assays in hiPSC derived cardiac myocytes is becoming commonplace due to the ease and availability of high speed/resolution optical imaging techniques. Typically, this uses voltage-sensitive dyes or genetically-encoded voltage indicators to measure action potentials and calcium wave propagation. Indeed, Guan and coworkers showed a two-fold increase in T50 (duration of recovery) of calcium transients in hiPSC-derived cardiac myocytes from DMD patients compared to healthy hiPSC derived cardiac myocytes [131]. In addition, Tsurumi et al. reported that the measurement of the fluorescent ratio (410/490 nm) of indo-1 demonstrated that the intracellular calcium concentration was much higher in cardiac myocytes differentiated from DMD-hiPSCs than in those differentiated from control-hiPSCs [132].

HiPSC-CMs represent a unique platform to study basic mechanisms of cardiomyopathies using a human cell-based system. The literature published in the past decade demonstrates the utility of patient-specific iPSCs in disease modeling of cardiomyopathy, and has provided unique insights into disease mechanisms. However, the hiPSC-CMs that are available today still largely represent an immature version of the adult cardiac myocyte, and thus have inherent limitations in the study of cardiovascular disease. Systems to induce greater maturation in hiPSC-CMs are being developed, but considerable work remains to be done to further advance this model system.

## 5. Currently Utilized Therapies for DMD Cardiomyopathy

### 5.1. Gene Therapy

Gene addition or gene correction therapies for DMD have gained considerable traction over the past few years. Multiple gene addition clinical trials for DMD are ongoing. These developments have been recently reviewed [133].

### 5.2. Drugs and Small Molecules

There is currently no cure for DMD, and established small molecule therapy is limited to reducing the symptoms and hindering the mechanisms of disease progression in the heart. One of the predominant small molecules that has been used as a treatment for DMD patients are corticosteroids. Corticosteroids ameliorate the skeletal muscle phenotype with marked improvement in muscle strength and function [134]. Other studies have demonstrated that the use of corticosteroids leads to the stabilization of pulmonary function, prolonged ambulation, and reduced prevalence of scoliosis [135]. Although corticosteroids exhibit more potent effects in skeletal muscle than cardiac muscle, studies show steroids to be protective in the dystrophic heart, with possible benefits including preserved ventricular function, reduction in fibrosis, and improved survival [136,137,138,139]. In addition, corticosteroids have been associated with delaying the onset of cardiomyopathy by 4% for each year of treatment [139]. As a result of these documented benefits to skeletal muscle, pulmonary, and cardiac function, a daily regimen with corticosteroids such as deflazacort and prednisone is currently the earliest and most widely used DMD therapy [140]. However, the prolonged use of this drug is not without controversy, with the primary side-effects including reduced bone density, increased adiposity, and increased muscle catabolism [141]. In addition, some concerns stem from preclinical studies providing evidence that steroids may worsen the progression of DMD in the mouse heart [142,143]. Nevertheless, DMD clinical studies are largely in agreement that steroids are likely to be protective in the dystrophic heart, with possible benefits including improved survival, preserved ventricular function, and reductions in fibrosis.

Angiotensin receptor blockers (ARBs) and angiotensin converting enzyme inhibitors (ACEIs) constitute another class of small molecules that have been used as a treatment for DMD patients [144]. Angiotensin II (AngII) and the AngII type 1 receptor (AT1R) exhibit many fundamental effects on the heart, including promoting fibrosis, ROS production, remodeling, and cardiomyocyte death [145,146,147]. ACE inhibitors are generally implemented when LV systolic dysfunction declines. These inhibitors prevent the conversion of angiotensin-I to angiotensin-II (Ang-II), thereby reducing circulating levels of Ang-II. When angiotensin-II is activated, it stimulates the adrenal cortex to secrete aldosterone, promoting fluid and sodium retention. Both angiotensin-II and aldosterone contribute to the formation of fibrosis and overgrowth of connective tissue in the heart and further complicate the myocardial fibrosis resulting from dystrophin deficiency in DMD patients [148]. Therefore, the use of ACE inhibitors, aldosterone antagonists, and angiotensin receptor blockers (ARBs) has become a significant therapeutic approach for dystrophic cardiomyopathy. ACE inhibitors are a drug class that is often used as the first line of therapy for general heart failure, and was the first drug to be used in trials to demonstrate improved cardiac function and survival among DMD patients [149]. ARBs are equally effective when compared directly to ACEIs and better tolerated by patients, but they are usually utilized as an alternative in cases of poor ACEI tolerance [150].

Beta-blockers are another class of small molecules that are regularly used for the treatment of acquired heart failure, where they are often combined with ACEIs to improve survival and reduce hospitalization rates [151]. Beta-blockers have been considered a candidate for cardiac-directed therapy in DMD aiming to limit β-AR effects. Beta-blockers act by interfering with beta-receptor binding by catecholamines, which leads to a reduction of sympathetic nervous system activity. They are therefore prescribed for arrhythmic patients and those with symptomatic but stable systolic dysfunction. It is known that combination therapy with ACE and β-adrenergic blocker agents improves the survival of patients with left ventricular dysfunction [152].

A retrospective study demonstrated that DMD patients have improvement in echocardiographic parameters, such as fractional shortening, sphericity index, and left ventricular ejection, after the administration of either ACE inhibitors alone or the combination of both ACE inhibitors and beta-blockers [153]. In a study conducted by Kajimoto and coworkers, patients with different types of muscular dystrophies were assigned to receive either an ACE inhibitor plus a beta-blocker (carvedilol) for at least 2 years or an ACE inhibitor alone (cilazapril or enalapril) for at least 3 years. They observed that the ACE inhibitor treatment alone maintained fractional shortening whereas the combination therapy provided a significant improvement in left ventricular fractional shortening [154]. Studies have also demonstrated that the use of ACE inhibitors in combination with beta -blockers in DMD patients reversed congestive heart failure signs and symptoms, delayed the progression of left ventricular dysfunction, and also improved systolic function [144]. Although some DMD patient studies indicate that beta-blockers have additive effects on cardiac function and survival compared with ACEIs alone, some studies using beta-blockers in DMD cardiomyopathy have shown little effect, making it unclear as to whether these drugs deliver a significant benefit [155,156].

Mineralocorticoid receptor antagonists, such as eplerenone and spironolactone are another class of small molecules that have been used for managing heart failure with low ejection fraction, and are often incorporated into treatment for DMD cardiomyopathy [11]. In a pre-clinical study of DMD cardiomyopathy, the combination of spironolactone and ACEI therapy showed protective effects in both skeletal and cardiac muscle [143]. A recent clinical trial demonstrated that DMD patients with preserved ejection fraction already receiving treatment with an ACEI or ARB showed modest but significant improvements in myocardial strain, ejection fraction, and chamber dilation with eplerenone treatment, compared to those without eplerenone [157].

## 6. Experimental Therapeutic Strategies to Improve Calcium Handling and Decrease Calcium Overload in DCM and DMD Cardiomyopathy

Although the mechanisms of cardiac dysfunction in DMD are complex and multifactorial, impaired calcium handling and calcium overload are key contributors to both early and late stage pathogenesis, as previously described. Currently utilized clinical therapies for DMD cardiomyopathy do not directly target calcium handling defects. Therefore, development of novel therapeutic strategies should focus on targeting various aspects of these pathways. Upstream targets include repair of the damaged sarcolemma and restoration of hyperactive stretch-activated channel (SAC) activity and RyR2 function. These targets will mitigate calcium overload from both the extracellular space and intracellular stores. Downstream targets include normalization of Serca2a/PLN activity and calcium cycling. These targets will mitigate the contraction and relaxation deficits characteristic of the dilated phenotype in late stage DMD cardiomyopathy. A summary of studies that specifically target various aspects of calcium mishandling and overload in cardiac tissue of muscular dystrophy models is shown in Table 2.

### 6.1. Membrane Stabilization

Membrane stabilization has been extensively studied as a therapeutic strategy for DMD cardiomyopathy in both small and large animal models [41,68] to mitigate sarcolemmal damage and calcium overload occurring from mechanical stress in the absence of dystrophin. Triblock copolymers, of which Poloxamer 188 (P188) has been most widely studied, consist of a hydrophobic polypropylene oxide (PPO) core, flanked by two hydrophilic polyethylene oxide (PEO) chains [63]. P188 is thought to insert into damaged membranes to provide stability until intrinsic repair mechanisms are able to restore integrity of the membrane [63]. P188 treatment of isolated dystrophic cardiac myocytes improved membrane compliance and decreased calcium influx and hypercontracture during passive physiological stretch [41]. *Ex vivo mdx* heart function was also improved after ischemia reperfusion injury with P188 treatment [159]. Finally, P188-treated *mdx* mice undergoing chronic isoproterenol stress had some improvements in *in vivo* cardiac function after two and four weeks [158], and a large animal model of DMD showed significant improvement in *in vivo* cardiac function after chronic P188 therapy [68]. The reader is referred to excellent recent reviews for more details on this topic [63,166].

Repairing damaged membranes using muscle-specific TRIM (tripartite motif) protein mitsugumin 53 (MG53) provides an additional avenue for targeting membrane instability in DMD. Recombinant MG53 administration to *mdx* mice improved skeletal muscle pathology and decreased damage after downhill treadmill running [167].

### 6.2. Stretch-Activated Channel Inhibition

In addition to micro-tears in the sarcolemma, increased expression and activity of stretch-activated non-selective ion channels (SACnsc) have been implicated in calcium influx and overload in muscular dystrophy. In skeletal muscle, the transient receptor potential channel (TRPC) family was identified as a therapeutic target for decreasing extracellular calcium entry in DMD. Inhibition of TRPC1 and TRPC4 expression with antisense oligonucleotides decreased calcium entry measured by patch-clamping in myofibers from *mdx* mice [168]. Further, transgenic expression of a dominant negative form of TRPC3 in *mdx* and *Scgd*^-/-^ mice decreased the dystrophic phenotype in skeletal muscle, including decreased fibrosis, serum CK, and occurrence of central nuclei [16]. In the heart, increased expression of TRPC1 has been implicated in the increased slow force response in *mdx* mice, caused by slow calcium influx leading to increased force production over several minutes of myocyte stretch [160]. Blockage of this channel with stretch-activated channel blocker GsMTx-4 decreased resting calcium concentration [160]. Further research is needed to explore whether decreased SAC activity or expression in the heart can delay or inhibit the dilated phenotype of DMD cardiomyopathy.

### 6.3. RyR2 Stabilization

RyR2 hypersensitivity has been implicated in calcium overload within the dystrophic cardiac myocyte [71]. Calstabin2 is a subunit of RyR2, which stabilizes the closed state of this channel [169]. Phosphorylation [169] or oxidative stress leading to nitrosylation [161] inhibits the association of calstabin2 with RyR2, increasing SR calcium leak. Treatment with N-acetyl cysteine to prevent nitrosylation or RyR2 stabilizer Rycal S107 prevented depletion of calstabin2 and decreased production of calcium sparks and depolarization in *mdx* cardiac myocytes [161]. Treatment of *mdx* mice with Rycal S107 also significantly reduced arrhythmias [161]. Similar studies in skeletal muscle found RyR1 stabilization to improve muscle strength, exercise tolerance, and muscle histopathology [58,170]. Whether long-term treatment to stabilize RyR2 in the heart can lead to improved cardiac outcomes in DMD requires further investigation.

### 6.4. Modulation of Serca2a/PLN

As a result of the central role of Serca2a and PLN in cardiac calcium cycling, their necessity in the maintenance of low diastolic calcium concentration, and the changes associated with their expression and activity in DCM, this complex has become an important experimental therapeutic target in both DCM and DMD-cardiomyopathy.

#### 6.4.1. Serca2a as a Target for DCM Therapy

Serca2a gene therapy has been studied extensively in a variety of heart failure models. Adenoviral gene transfer of Serca2a in isolated cardiac myocytes from failing human hearts improved peak height of contraction and relaxation rate, which could be explained by increased calcium peak height and decay rate [171]. Multiple small animal models of heart failure have also been used to study the effectiveness of Serca2a gene therapy. Aortic-banded rats with adenoviral gene delivery of Serca2a had improved systolic and diastolic function measured by *in vivo* hemodynamics [172], increased survival rate [173], normalized energetics (PCr:ATP), and more efficient oxygen utilization compared to aortic-banded rats without Serca2a treatment [173,174]. Lentiviral-mediated Serca2a gene delivery after myocardial infarction in rats led to improvement in left ventricular systolic and diastolic dimensions and fractional shortening measured by echocardiography, improved hemodynamic measurements of systolic and diastolic function, and increased survival rate [175]. Two large animal models have also been used to study the effectiveness of Serca2a gene delivery via adeno-associated viral (AAV) gene transfer. In a swine model of mitral regurgitation, AAV delivery of Serca2a led to improved LV systolic and diastolic dimensions, left ventricular ejection fraction, and +dP/dt two months after gene delivery. AAV2/1-Serca2a in sheep with rapid pacing-induced heart failure also had improvements in LV dimensions, as well as increased systolic function and improvements in the pressure-volume relationship [176,177].

As a result of its therapeutic benefit in pre-clinical models of heart failure, Serca2a gene therapy has been tested in two randomized, placebo-controlled clinical trials using intracoronary delivery of AAV1 in advanced heart failure patients. In the first trial, patients that received the highest dose of Serca2a (1 × 10^13^ DNase resistant particles) showed improvement in pre-defined clinical endpoints including symptomatic, functional, biomarker, and LV function and remodeling abnormalities after six months [178]. The high-dose group also had an 82% decrease in recurrent cardiovascular events after three years of follow-up [179]. This study was followed by a larger multi-center trial of 250 heart failure patients using the high dose of Serca2a. Unlike the initial trial, there was no difference between treatment and control groups in either recurrent or terminal clinical events [180]. The basis for the discrepancy in results between the two studies is uncertain. A small number of heart tissue samples revealed low vector DNA copy number. Additionally, there were differences between the two trials in the number of total viral particles (including empty capsids) delivered [180].

#### 6.4.2. PLN as a Target for DCM Therapy

As the major regulator of Serca2a, PLN has also been studied for its potential role to mitigate the calcium cycling deficits contributing to DCM. Early studies found PLN overexpression or deficiency decreased or increased calcium cycling and Serca2a calcium sensitivity, respectively, and β-adrenergic signaling was important in determining the level of PLN inhibition of Serca2a [181,182]. Increasing the ratio of PLN to Serca2a in a transgenic mouse model with 2-fold overexpression of PLN decreased peak height of contraction as well as slowed relaxation rate. This was accompanied by similar decreases in calcium transient peak height and decay rate. Depressed *in vivo* function measured by echocardiography was also present, and this was alleviated by isoproterenol treatment [181]. Contrary to PLN overexpression, PLN deficiency increases calcium cycling and cardiac function. Working heart preparations from PLN-deficient mice exhibited both increased systolic and diastolic function, which was not further enhanced by beta-adrenergic stimulation [182]. Increased calcium cycling and subsequent increases in contraction and relaxation function with PLN depletion are dose dependent, with PLN (+/-) mice exhibiting cardiac function at levels between WT and PLN (-/-) animals [183].

These early studies solidifying the inhibitory role of PLN on calcium cycling led to the hypothesis that PLN inhibition may ameliorate the depressed calcium cycling characteristic of DCM. This hypothesis has been tested in multiple models of DCM. In the MLP-deficient mouse [92], PLN deficiency decreased dilation and ultrastructural abnormalities and increased heart function measured by *in vivo* hemodynamics and echocardiography. These improvements could be explained by increased calcium cycling measured in isolated cardiac myocytes [184]. A pseudo-phosphorylated mutant of PLN (S16E) mimics the conformational change occurring in PLN after PKA phosphorylation at serine-16, decreasing interaction with Serca2a. *In vivo* rAAV gene transfer of S16E-PLN improved multiple measures of heart function in a post-myocardial infarction rat model, including dilation, heart size, ejection fraction and E/A ratio measured by echocardiography at 2 and 6 months post-MI. Additionally, multiple hemodynamic measurements were improved compared to infarcted rats without AAV treatment [185]. Similar improvements in heart function were observed in a large animal model of heart failure with S16E-PLN gene transfer. Sheep undergoing four weeks of pacing stress, followed by *in vivo* percutaneous cardiac recirculation-mediated gene delivery of S16E-PLN recovered hemodynamic function after two weeks, whereas control animals continued to have worsening heart failure [186]. Another approach to inhibition of PLN is to inhibit Phosphatase-1 (PP1), which dephosphorylates PLN and causes it to bind and inhibit Serca2a. Using AAV-9 gene delivery of a short-hairpin RNA against PP1beta with a B-type natriuretic peptide (BNP)-promoter created a heart-failure inducible expression system which was tested in the MLP-deficient mouse. This system increased PLN phosphorylation, which was accompanied by decreased cardiac remodeling and improved fractional shortening and hemodynamic function three months after gene transfer [187].

#### 6.4.3. Serca2a and PLN in Muscular Dystrophy-Associated Cardiomyopathy

Although most research has focused on the role of Serca2a/PLN in DCM in general, a number of studies have looked specifically at the cardiomyopathy occurring in models of muscular dystrophy. Serca2a gene expression is decreased in mice with DMD cardiomyopathy [188], suggesting the Serca2a/PLN complex may also be an important therapeutic target for DCM occurring in DMD.

Two studies examined the effect of Serca1 overexpression in skeletal muscle, and one study looked at Serca2a overexpression in the heart of different mouse models of muscular dystrophy. Crossing Serca1 transgenic mice with *mdx*, *mdx:utr*^-/-^, and *Sgcd*^-/-^ mice, resulting in a 1.5–4-fold overexpression of Serca1, decreased CK release [189,190], reduced Evan’s Blue Dye (EBD) uptake [189] and decreased fibrosis [189], suggesting decreased muscle damage. This was attributed to the ability of Serca1 overexpression to improve calcium handling in isolated myocytes [189]. The result of Serca1 overexpression led to restored treadmill running capability [189] and decreased percent torque loss after eccentric contraction-induced injury [190]. Serca2a overexpression in the aged *mdx* heart was examined using AAV-9 gene delivery. In this study, Serca2a was unable to reverse fibrosis, but did result in some improvement in electrocardiographic abnormalities [162].

PLN inhibition has also been tested in the context of muscular-dystrophy associated cardiomyopathy. The BIO14.6 hamster is a model of limb-girdle muscular dystrophy and exhibits a progressive cardiomyopathy phenotype beginning by about 5 weeks of age [191]. Recombinant AAV gene delivery of S16E-PLN in 5-6 week old BIO14.6 hamsters improved calcium cycling in isolated SR vesicles. This resulted in increased fractional shortening and improved maximum and minimum LV dP/dt at both 5 and 28 weeks post-gene transfer compared to BIO14.6 hamsters without AAV treatment [163]. Another study in the BIO14.6 hamster model used adenoviral gene delivery of an antibody targeted against PLN. Just over 50% of myocytes were infected with the virus, which led to short-term improvement in both echocardiographic and hemodynamic markers of systolic and diastolic function. This was accompanied by improved contractility and calcium handling in isolated myocytes, and increased SR calcium reuptake in whole heart homogenates [164]. Additionally, inhibition of PP1 via gene delivery of Inhibitor-2 in the BIO14.6 hamster resulted in beneficial effects on cardiac dimensions and fractional shortening, as well as improved hemodynamic measurements, decreased fibrosis, and improved survival [165].

Similar to PLN, sarcolipin (SLN) is an inhibitor of Serca. SLN expression is increased in heart muscle of *mdx:utr*^-/-^ mice, dystrophic dogs and human patients with DMD. Deletion of either one or both alleles of SLN in *mdx:utr*^-/-^ mice extended the lifespan of these animals, as well as improved heart function (ejection fraction and fractional shortening). Improved function was attributed to decreased left ventricular internal diameter in diastole (LVIDd) and decreased fibrotic and necrotic tissue. Although calcium handling was not measured in heart tissue in this study, skeletal muscle calcium reuptake was increased as a result of reduced SLN expression [192].

**Figure 4:**
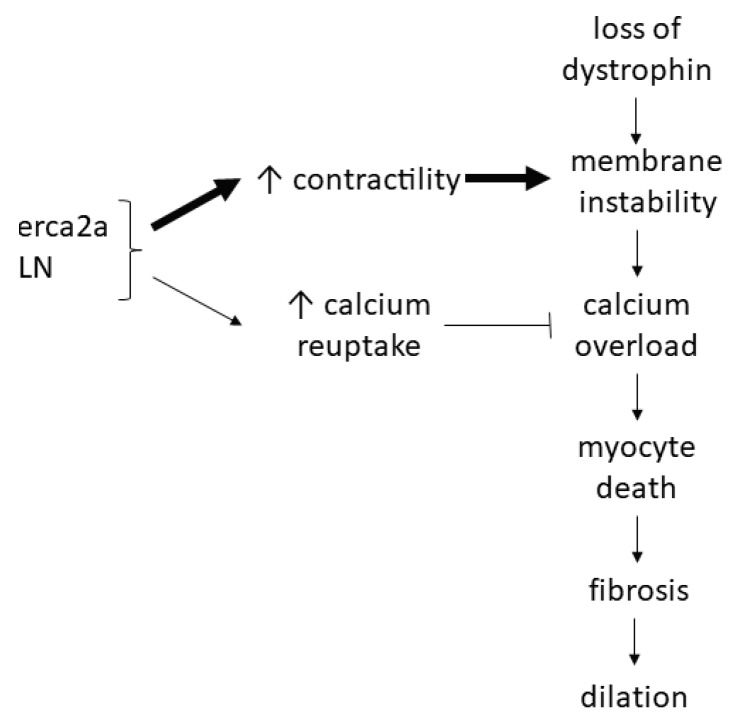
Effects of increased calcium cycling on cardiac myocytes with dystrophin deficiency. Loss of dystrophin destabilizes the sarcolemma and leads to calcium mishandling and overload. Increasing calcium cycling via modulation of Serca2a/PLN function increases calcium uptake into the SR, which could decrease cytosolic calcium concentration. However, increased calcium cycling also increases contractility, which could subsequently cause increased membrane damage and exacerbate calcium overload. In the context of dystrophin deficiency, phospholamban (PLN) ablation led to increased membrane damage and worsened cardiomyopathy [19].

As a result of the extensive pre-clinical literature showing beneficial effects of Serca2a overexpression or PLN inhibition on calcium handling and heart failure outcomes in multiple models of heart failure, including muscular dystrophy-associated cardiomyopathy, our laboratory hypothesized that PLN ablation would improve calcium handling and subsequently improve cardiac function in the *mdx* mouse. Isolated cardiac myocytes from these mice did indeed show enhanced contractility and faster relaxation, which was accompanied by increased calcium transient peak height and decay rate. However, *in vivo* echocardiography revealed severe dilation and decreased systolic and diastolic function. Histological analysis revealed significantly more fibrotic development and EBD uptake, indicating sarcolemma integrity was more severely compromised with PLN ablation [19]. It was concluded that, although PLN ablation improved calcium cycling in isolated myocytes, which could potentially decrease the risk of calcium overload, increased contractile function likely placed additional stress on an already compromised sarcolemma. This likely led to even more extensive membrane damage than occurs with dystrophin deficiency alone [19] (Figure 4).

The results of this study are in contrast to others discussed above [162,163,164,165]. One potential contributor to these differences is the level of PLN inhibition. In the context of muscular dystrophy models, one study overexpressed Serca2a [162], and others have inhibited PLN to varying degrees through delivery of a pseudophosphorylated PLN [163], an antibody against PLN [164], or inhibitor-2 [165]. Although complete ablation of PLN improved cardiomyopathy in a DCM model [184], this was not the case in *mdx* mice, highlighting key differences mechanistically between the pathophysiology of muscular dystrophy-associated DCM and other causes of DCM. PLN knockout mice have significantly increased calcium cycling leading to increased contractile function [182,183], which likely increased sarcolemmal stress. Additionally, PLN knockout mice are not responsive to β-adrenergic signaling [182] and therefore have very little cardiac reserve. PLN knockout mice also have an increased ATP utilization and oxygen consumption [193], potentially increasing oxidative stress and potentiating membrane damage in the context of muscular dystrophy, which exhibits decreased endogenous reducing capability [66]. Finally, gene deletion or transgenic expression of genes may have unknown compensatory effects on development which cannot be controlled for. Whether small increases in Serca2a expression or partial inhibition of PLN in *mdx* mice would yield different results needs to be the focus of future studies.

#### 6.4.4. Caution with Serca2a/PLN Therapy for Cardiomyopathy

Although many pre-clinical models of heart failure, including those of muscular dystrophy-associated cardiomyopathy, show significant improvement in cardiac morphology, calcium handling, contractile function, and survival with decreased PLN inhibition of Serca2a, caution should be taken when translating this to human disease. Two mutations leading to loss of PLN function have been identified in humans. A point mutation (T116G) resulting in a premature stop codon and a nonfunctional PLN protein (Leu39-stop) leads to severe DCM requiring transplantation at a young age [194]. Co-expression of Leu39-stop PLN and Serca2a in HEK293 cells revealed this truncated PLN protein was unable to decrease Serca2a affinity for calcium, and gene transfer of Leu39-stop PLN in isolated cardiac myocytes had no effects on calcium cycling or contraction/relaxation kinetics [194]. A C→T missense mutation at nucleotide 25 of the PLN gene encodes an Arg→Cys substitution (R9C) in the cytosolic domain, which also results in DCM and early death [195]. Similar to the Leu39-stop mutation, R9C did not inhibit calcium uptake in HEK293 cells [195]. Further study of this mutation revealed that R9C stabilizes the pentamer conformation of the protein, making it unavailable to inhibit Serca2a [196]. Indeed, acute expression of R9C via adenoviral gene transfer in rabbit cardiac myocytes revealed an increase in contractility and relaxation kinetics, with a concomitant increase in calcium peak height and decay rate [197]. Additionally, R9C transfected cardiac myocytes showed decreased responsiveness to beta-adrenergic stimulation [197]. Finally, patients with specific polymorphisms in alpha2c and beta1 adrenergic receptors, which leads to increased release and sensitivity to norepinephrine, have an odds ratio of 10.11 of developing heart failure compared to patients without these polymorphisms [198]. Chronically increased adrenergic stimulation increases load on the heart and decreases cardiac reserve, both of which also occur with PLN inhibition.

Key differences in cardiac physiology and calcium handling exist between mice and humans, which could account for the divergent outcomes. Approximately 90% of calcium reuptake in mice occurs via Serca2a and only 10% occurs via the sarcolemmal Na^+^/Ca^2+^ exchanger. In contrast, approximately one-third of calcium is exported from the cytosol via the Na^+^/Ca^2+^ exchanger in humans. Additionally, resting heart rate is approximately 10-fold lower in humans compared to mice, and the human heart has a greater variability in heart rate response to physiological stress. Finally, humans have a higher cardiac reserve allowing for increased SR calcium uptake and calcium load during physiological stress. Reducing cardiac reserve in humans by inhibiting PLN or increasing Serca2a may therefore have a much different outcome compared with a similar inhibition in rodents [56,199]. This was demonstrated in the CUPID 2 trial, where Serca2a gene delivery was not successful in meeting beneficial clinical outcomes in heart failure patients [180].

### 6.5. Calcium Buffering

An alternative and energetically neutral approach to improving calcium reuptake and relaxation rate is to introduce expression of *de novo* calcium buffers into cardiac myocytes. The most well-studied calcium buffer in this context is parvalbumin. Parvalbumin (Parv) is ~12 kDa EF-hand calcium/magnesium binding protein naturally expressed in fast-twitch muscle in order to aid in fast relaxation by buffering calcium away from myofilaments [200,201]. Parvalbumin proteins contain two 12 amino acid EF-hand cation binding loops with binding affinities ranging from K_Ca_^2+^ = 10^7^−10^9^ M^−1^ and K_Mg_^2+^ = 10^3^−10^5^ M^−1^, for calcium and magnesium, respectively [202,203]. In a resting myocyte, magnesium concentration is ~1 mM and calcium concentration is 10–100 nM, resulting in magnesium occupancy of the EF-hand loops. During contraction, calcium concentration increases, which causes calcium to displace magnesium in the binding loops. Calcium binding to Parv buffers calcium away from myofilaments to aid in rapid relaxation. As calcium is taken back up into the SR during relaxation, cytosolic calcium concentration falls, resulting in magnesium reoccupying the EF-hand cation binding loops. The ability of Parv to bind both magnesium and calcium is important in its function as a delayed calcium buffer. Calcium concentration must be sufficiently increased to induce magnesium dissociation from the EF-hand binding loops. This delay in calcium binding enables calcium to first bind to myofilaments and facilitate contraction of the myocyte before binding to Parv [203].

The ability of Parv to facilitate fast relaxation in the heart has been tested in numerous cell and animal models of diastolic dysfunction. Adenoviral gene transfer of Parv into isolated cardiac myocytes from hypothyroid rats [204], senescent rats [205], and Dahl salt-sensitive rats [206] hastened relaxation by increasing the rate of calcium decay. *In vivo* studies revealed gene delivery of Ad-Parv improved short-term hemodynamic measurements of relaxation, including -dP/dt and time to 50% and 90% pressure decay in hypothyroid rats [207], and decreased tau, a load independent measure of diastolic dysfunction, in senescent rats [208,209]. One advantage to a calcium buffering approach for improved relaxation over increasing Serca2a activity is the energetic efficiency. Mathematical modeling studies indicate Serca2a overexpression leads to a higher peak and total ATP consumption compared to *de novo* Parv expression, which results in a more even distribution of ATP consumption over the course of the contractile cycle [210]. Additionally, Parv expression in cardiac myocytes preserves beta-adrenergic function, which is blunted with increased Serca2a function [211] or PLN inhibition [182].

One drawback to using wild-type Parv for improved relaxation in the heart is the calcium/magnesium binding affinities are not optimized for the relatively slow contractile cycle of the heart. Although Parv is a delayed calcium buffer, requiring magnesium removal from the EF-hand cation binding sites before calcium can bind, the kinetics are optimized for fast-twitch muscle. This results in WT-Parv binding calcium too early in the contractile cycle of cardiac myocytes and inhibiting maximal contractility [206,212]. This is contraindicated in the context of dilated cardiomyopathy, which is characterized by both decreased contractile function and slowed relaxation. A potential solution to this problem with WT-Parv is to genetically modify the EF-hand cation binding site to have optimal binding affinities for the kinetics of the human heart. It was hypothesized and confirmed with mathematical modeling that increasing magnesium affinity and slowing magnesium off-rate even further than WT-Parv would restrict the buffering of calcium to diastole and prevent premature truncation of contraction [210].

To test this hypothesis in myocytes, two genetically modified parvalbumin proteins have been developed. Both involve substitutions of the highly conserved glutamate at residue 12 of the EF-hand cation binding site, one with glutamine (E101Q) [213] and one with aspartate (E101D) [214]. These substitutions eliminate the 7th coordinating oxygen preferred by calcium, resulting in both an increase in magnesium affinity and a decrease in calcium affinity, further delaying the buffering of calcium compared to WT-Parv. Adenoviral gene transfer of these modified parvalbumins increased relaxation rate and unexpectedly also increased contraction amplitude in isolated myocytes from rat (E101Q and E101D), rabbit (E101Q) and canine (E101Q). Additionally, ParvE101Q improved contractility and relaxation in multiple models of heart failure, including thapsigargin-treated rabbit myocytes, failing myocytes from dogs, and *in vivo* hemodynamic function of inducible Serca2a knock-out mice [213,214].

As a result of its primary role in increasing relaxation, most studies with Parv have been done using models of diastolic dysfunction. This is because, as a calcium buffer, the main contribution of Parv in the context of the failing heart has been thought to be sequestration of calcium to enhance relaxation in diastole. In particular, the decreased contractility characteristic of WT-Parv-treated myocytes would be contraindicated for the already compromised contractility in DCM. Whether or not modified parvalbumins could serve to both improve relaxation and increase contractility in models of DCM is unknown and warrants further investigation, particularly because Parv both preserves beta-adrenergic function and is an energetically neutral approach to improving function in the energetically compromised failing heart [215]. Calcium buffering may potentially have a beneficial role in the cardiomyopathy of DMD. Increased diastolic calcium resulting from calcium influx through SACs, sarcolemmal micro-tears, and increased RyR2 leak could theoretically be mitigated through introduction of a calcium buffering system. The localization of these buffers within the myocyte, as well as the buffering capacity and calcium binding kinetics are all factors that need to be optimized and tested when considering this approach in the context of DMD cardiomyopathy.

## 7. Conclusions

Cardiomyopathy is a significant clinical feature of DMD, with nearly all patients exhibiting cardiac dysfunction by their teens [13]. Improved clinical management of musculoskeletal and respiratory issues in DMD patients has uncovered cardiomyopathy as a significant contributor to morbidity and mortality [13]. The underlying pathology is complex, owing to the multiple functions of dystrophin in the cardiac myocyte, but calcium overload and mishandling due to membrane instability is a key mechanistic contributor to disease onset and progression. Currently utilized clinical therapies for DMD cardiomyopathy do not specifically target calcium handling defects and come with significant side effects. Gene therapeutic strategies to correct calcium handling defects show some promise in experimental models of DCM, but more work must be done to understand the potential benefits and risks of these strategies in models specific to DMD-related cardiomyopathy. Of note, increasing calcium cycling in the context of the membrane instability characteristic of DMD may exacerbate disease progression [19]. Continued work to understand the mechanistic underpinnings of DMD cardiomyopathy, specifically related to calcium handling, will enable a more targeted approach to therapeutic development for this disease.

## Figures and Tables

**Table 1:** Summary of model systems to study dilated cardiomyopathy (DCM) and Duchenne muscular dystrophy (DMD)-cardiomyopathy.

Model System	Strategy	Cardiac Phenotype
Rodent		
Muscle LIM protein (MLP) null mice [92]	Deletion of MLP (actin-associated cytoskeletal protein)	Anatomical and physiological hallmarks of human DCM
Desmin-deficient mice [91,93]	Desmin knockout mice	Severe loss of overall myocardial architecture by degeneration and calcification
Surgical interruptions of coronary arteries [94,95]	Produce myocardial infarction through permanent coronary ligation or re-perfused infarction	DCM phenotype progressively develops post-infarction
Doxorubicin or isoproterenol [96,97,98,99]	Toxic drug-mediated cardiomyopathy	Dose-dependent dilated phenotype and overt heart failure over time owing to severe myocardial injury and cell death
*mdx* mice [100,101]	Nonsense point mutation in exon 23 preventing dystrophin expression	Moderate DCM and functional cardiac impairment, progressive with age
Utrophin knockout mdx mice [102,103]	Crossing *mdx* mice to the utrophin-null background	Severe cardiomyopathy. Displays physiological indicators of end-stage heart failure
Large animals		
Dogs, pigs and sheep [104,105,106,107,108]	Myocardial infarction, coronary micro-embolization, pacing-induced tachycardia, and toxic injury	DCM phenotype progressively develops post-infarction
Golden retriever muscular dystrophy (GRMD) animal model of DMD [68,109,110]	Spontaneous splice site mutation in the *DMD* gene. Single nucleotide change that leads to exon skipping and an out-of-frame *DMD* transcript.	Prominent cardiac lesions present as early as 6 months of age, with ECG abnormalities present at 1 year and profound myocardial contractile abnormalities by 20 months
Human iPSCs		
iPSCs-CMs [111]	iPSCs-CMs derived from a member of a family with DCM carrying a heterozygous mutation in cardiac troponin T	iPSC-derived cardiomyocytes from DCM patients recapitulated to some extent the morphological and functional phenotypes of familial DCM with inherited mutation in troponin T
iPSCs-CMs [112]	Patient-specific DCM iPSC generated from a single member of a family with an autosomal dominant nonsense mutation (p.R225X) in exon 4 of the lamin A/C (LMNA) gene	iPSC-CMs showed morphologic changes, including a higher prevalence of nuclear bleb formation, micronucleation, as well as nuclear senescence and cellular apoptosis
iPSC-CMs [113]	iPSC-CMs derived from a patient with dilated cardiomyopathy with a novel heterozygous mutation of p.A285V codon conversion on exon 4 of the desmin gene	iPSC-CMs provided histologic and functional confirmation that the candidate gene variant detected by whole exome sequencing was responsible for the disease
iPSCs-CMs [114]	iPSC-CMs from DMD patients and healthy control	*In vitro* model that manifests the major phenotypes of DCM in DMD patients

MLP: muscle LIM-protein; ECG: electrocardiogram; iPSCs-CMs: human induced pluripotent stem cells-derived *cardiac myocytes.*

**Table 2:** Summary of research investigating experimental therapeutic strategies for calcium mishandling and overload in muscular dystrophy cardiomyopathy.

Target	Therapy	Model	Major Findings
Sarcolemma [41,68,158,159]	Copolymer –based membrane stabilizers	*mdx* mice dysferlin KO mice GRMD canine	↓ Myocyte Ca^2+^ influx/hypercontracture ↓ Stress-induced functional deficits (acute and chronic) ↓ Fibrosis, serum cTnI, LV remodeling ↓ *Ex vivo* ischemia/reperfusion injury
Stretch-Activated Channels [160]	GsMTX-4	*mdx* mice	↓ Myocyte resting Ca^2+^ concentration
Ryanodine Receptor [161]	N-acetyl cysteine Rycal S107	*mdx* mice	↓ Myocyte resting Ca^2+^ concentration ↓ Myocyte RyR2 Ca^2+^ leak ↓ Arrhythmias
Serca2a [162]	AAV-9 Serca2a	*mdx* mice	Normalized ECG measurements
Phospholamban [19,163,164,165]	AAV S16E-PLN Adenovirus anti-PLN antibody Adenovirus inhibitor-2 PLN-KO	BIO14.6 hamster *mdx* mice	PLN inhibition in BIO14.6 hamsters • ↑ Ca^2+^ reuptake in SR vesicles • ↑ Myocyte contractility and Ca^2+^ handling • ↑ LV systolic and diastolic function • ↓ Fibrosis • ↑ Survival PLN ablation in *mdx* mice • ↑ Myocyte contractility and Ca^2+^ handling • ↓ LV systolic and diastolic function • ↑ EBD uptake • ↑ Fibrosis

KO: knockout; cTnI: cardiac troponin I; LV: left ventricle; RyR2: ryanodine receptor 2; AAV: adeno-associated virus; ECG: electrocardiogram; PLN: phospholamban; EBD: Evan’s Blue Dye.

## References

[1] World Health Organization (2017). World Heath Statistics 2017: Monitoring Health for the SDGs, Sustainable Development Goals.

[2] Benjamin E.J., Muntner P., Alonso A., Bittencourt M.S., Callaway C.W., Carson A.P., Chamberlain A.M., Chang A.R., Cheng S., Das S.R. (2019). Heart Disease and Stroke Statistics-2019 Update: A Report From the American Heart Association. Circulation.

[3] Jefferies J.L., Towbin J.A. (2010). Dilated cardiomyopathy. Lancet.

[4] Towbin J.A. (2014). Inherited cardiomyopathies. Circ. J..

[5] Romitti P.A., Zhu Y., Puzhankara S., James K.A., Nabukera S.K., Zamba G.K.D., Ciafaloni E., Cunniff C., Druschel C.M., Mathews K.D. (2015). Prevalence of duchenne and becker muscular dystrophies in the United States. Pediatrics.

[6] Gao X., Shen X., Dong X., Ran N., Han G., Cao L., Gu B., Yin H.F. (2015). Peptide nucleic acid promotes systemic dystrophin expression and functional rescue in dystrophin-deficient mdx mice. Mol. Ther. Nucleic Acids.

[7] Emery A.E.H. (2002). The muscular dystrophies. Lancet.

[8] Simonds A.K., Muntoni F., Heather S., Fielding S. (1998). Impact of nasal ventilation on survival in hypercapnic Duchenne muscular dystrophy. Thorax.

[9] Porte P. (1999). Clinical indications for noninvasive positive pressure ventilation in chronic respiratory failure due to restrictive lung disease, COPD, and nocturnal hypoventilation—A consensus conference report. Chest.

[10] Eagle M., Baudouin S.V., Chandler C., Giddings D.R., Bullock R., Bushby K. (2002). Survival in Duchenne muscular dystrophy: Improvements in life expectancy since 1967 and the impact of home nocturnal ventilation. Neuromuscul. Disord..

[11] Kamdar F., Garry D.J. (2016). Dystrophin-Deficient Cardiomyopathy. J. Am. Coll. Cardiol..

[12] Shirokova N., Niggli E. (2013). Cardiac phenotype of Duchenne Muscular Dystrophy: Insights from cellular studies. J. Mol. Cell. Cardiol..

[13] Nigro G., Comi L.I., Politano L., Bain R.J.I. (1990). The incidence and evolution of cardiomyopathy in Duchenne muscular dystrophy. Int. J. Cardiol..

[14] Petrof B.J., Shrager J.B., Stedman H.H., Kelly A.M., Sweeney H.L. (1993). Dystrophin protects the sarcolemma from stresses developed during muscle contraction. Proc. Natl. Acad. Sci. USA.

[15] Sandri M., Podhorska-Okolow M., Geromel V., Rizzi C., Arslan P., Franceschi C., Carraro U. (1997). Exercise Induces Myonuclear Ubiquitination and Apoptosis in Dystrophin-deficient Muscles of Mice. J. Neuropathol. Exp. Neurol..

[16] Millay D.P., Goonasekera S.A., Sargent M.A., Maillet M., Aronow B.J., Molkentin J.D. (2009). Calcium influx is sufficient to induce muscular dystrophy through a TRPC-dependent mechanism. Proc. Natl. Acad. Sci. USA.

[17] Peterson J.M., Wang D.J., Shettigar V., Roof S.R., Canan B.D., Bakkar N., Shintaku J., Gu J.M., Little S.C., Ratnam N.M. (2018). NF-κB inhibition rescues cardiac function by remodeling calcium genes in a Duchenne muscular dystrophy model. Nat. Commun..

[18] Williams I.A., Allen D.G. (2007). Intracellular calcium handling in ventricular myocytes from mdx mice. Am. J. Physiol. Heart Circ. Physiol..

[19] Law M.L., Prins K.W., Olander M.E., Metzger J.M. (2018). Exacerbation of dystrophic cardiomyopathy by phospholamban deficiency mediated chronically increased cardiac Ca^2+^ cycling in vivo. Am. J. Physiol. Heart Circ. Physiol..

[20] Fanchaouy M., Polakova E., Jung C., Ogrodnik J., Shirokova N., Niggli E. (2009). Pathways of abnormal stress-induced Ca^2+^ influx into dystrophic mdx cardiomyocytes. Cell Calcium.

[21] Li Z., Li Y., Zhang L., Zhang X., Sullivan R., Ai X., Szeto C., Cai A., Liu L., Xiao W. (2017). Reduced Myocardial Reserve in Young X-Linked Muscular Dystrophy Mice Diagnosed by Two-Dimensional Strain Analysis Combined with Stress Echocardiography. J. Am. Soc. Echocardiogr..

[22] Li Y., Zhang S., Zhang X., Li J., Ai X., Zhang L., Yu D., Ge S., Peng Y., Chen X. (2014). Blunted cardiac beta-adrenergic response as an early indication of cardiac dysfunction in Duchenne muscular dystrophy. Cardiovasc. Res..

[23] Mcnally E.M., Mestroni L. (2017). Dilated cardiomyopathy: Genetic determinants and mechanisms. Circ. Res..

[24] Bers D.M. (2002). Cardiac excitation-contraction coupling. Nature.

[25] Townsend D.W., Yasuda S., Chamberlain J., Metzger J.M. (2009). Cardiac Consequences to Skeletal Muscle-Centric Therapeutics for Duchenne Muscular Dystrophy. Trends Cardiovasc. Med..

[26] Berko B.A., Swift M. (1987). X-Linked Dialted Cardiomyopathy. N. Engl. J. Med..

[27] Towbin J.A., Hejtmancik J.F., Brink P., Gelb B., Zhu X.M., Chamberlain J.S., McCabe E.R.B., Swift M. (1993). X-linked dilated cardiomyopathy: Molecular genetic evidence of linkage to the Duchenne muscular dystrophy (dystrophin) gene at the Xp21 locus. Circulation.

[28] Eagle M., Bourke J., Bullock R., Gibson M., Mehta J., Giddings D., Straub V., Bushby K. (2007). Managing Duchenne muscular dystrophy The additive effect of spinal surgery and home nocturnal ventilation in improving survival. Neuromuscul. Disord..

[29] Gordon A.M., Homsher E., Regnier M. (2000). Regulation of Contraction in Striated Muscle. Physiol. Rev..

[30] Kamisago M., Sapna S., DePalma S., Solomon S., Sharma P., McDonough B., Smoot L., Mullen M.P., Woolf P.K., Wigle D. (2000). Mutations in sarcomere protein genes as a cause of dilated cardiomyopathy. N. Engl. J. Med..

[31] McNally E.M., Golbus J.R., Puckelwartz M.J. (2013). Genetic mutations and mechanisms in dilated cardiomyopathy. J. Clin. Investig..

[32] Gomes A.V., Potter J.D. (2004). Molecular and Celluar Aspects of Troponin Cardiomyopathies. Ann. N. Y. Acad. Sci..

[33] Wolff M.R., Buck S.H., Stoker S.W., Greaser M.L., Mentzer R.M. (1996). Myofibrillar calcium sensitivity of isometric tension is increased in human dilated cardiomyopathies: Role of altered β-adrenergically mediated protein phosphorylation. J. Clin. Investig..

[34] Nakano S.J., Walker J.S., Walker L.A., Li X., Du Y., Miyamoto S.D., Sucharov C.C., Garcia A.M., Mitchell M.B., Ambardekar A.V. (2019). Increased myocyte calcium sensitivity in end-stage pediatric dilated cardiomyopathy. Am. J. Physiol. Heart Circ. Physiol..

[35] Van Der Velden J., De Jong J.W., Owen V.J., Burton P.B.J., Stienen G.J.M. (2000). Effect of protein kinase A on calcium sensitivity of force and its sarcomere length dependence in human cardiomyocytes. Cardiovasc. Res..

[36] Memo M., Leung M.C., Ward D.G., Dos Remedios C., Morimoto S., Zhang L., Ravenscroft G., McNamara E., Nowak K.J., Marston S.B. (2013). Familial dilated cardiomyopathy mutations uncouple troponin i phosphorylation from changes in myofibrillar Ca^2+^ sensitivity. Cardiovasc. Res..

[37] Messer A.E., Marston S.B. (2014). Investigating the role of uncoupling of troponin I phosphorylation from changes in myofibrillar Ca^2+^-sensitivity in the pathogenesis of cardiomyopathy. Front. Physiol..

[38] Chang A.N., Potter J.D. (2005). Sarcomeric protein mutations in dilated cardiomyopathy. Heart Fail. Rev..

[39] Mestroni L., Brun F., Spezzacatene A., Sinagra G., Taylor M.R. (2014). Genetic Causes of Dilated Cardiomyopathy. Prog. Pediatr Cardiol.

[40] McNally E.M. (2012). Broken giant linked to heart failure. Nature.

[41] Yasuda S., Townsend D.W., Michele D.E., Favre E.G., Day S.M., Metzger J.M. (2005). Dystrophic heart failure blocked by membrane sealant poloxamer. Nature.

[42] Barresi R., Di Blasi C., Tiziana N., Brugnoni R., Vitali A., Felisari G., Salandi A., Daniel S., Cornelio F., Morandi L. (2000). Disruption of heart sarcoglycan complex and severe cardiomyopathy caused by β sarcoglycan mutations. J. Med. Genet..

[43] Tsubata S., Bowles K.R., Vatta M., Zintz C., Titus J., Muhonen L., Bowles N.E., Towbin J.A. (2000). Mutations in the human δ-sarcoglycan gene in familial and sporadic dilated cardiomyopathy. J. Clin. Investig..

[44] Shaw T., Elliott P., McKenna W.J. (2002). Dilated cardiomyopathy: A genetically heterogeneous disease. Lancet.

[45] Sequeira V., Nijenkamp L.L.A.M., Regan J.A., Van Der Velden J. (2014). The physiological role of cardiac cytoskeleton and its alterations in heart failure. Biochim. Biophys. Acta Biomembr..

[46] Heydemann A., McNally E.M. (2007). Consequences of Disrupting the Dystrophin-Sarcoglycan Complex in Cardiac and Skeletal Myopathy. Trends Cardiovasc. Med..

[47] Weintraub R.G., Semsarian C., Macdonald P. (2017). Dilated cardiomyopathy. Lancet.

[48] Kearney M.T., Cotton J.M., Richardson P.J., Shah A.M. (2001). Viral myocarditis and dilated cardiomyopathy: Mechanisms, manifestations, and management. Postgrad. Med. J..

[49] Barnabei M.S., Sjaastad F.V., Townsend D.W., Bedada F.B., Metzger J.M. (2015). Severe dystrophic cardiomyopathy caused by the enteroviral protease 2A-mediated C-terminal dystrophin cleavage fragment. Sci. Transl. Med..

[50] Parodi O., De Maria R., Oltrona L., Testa R., Sambuceti G., Roghi A., Merli M., Belingheri L., Accinni R., Spinelli F. (1993). Myocardial blood flow distribution in patients with ischemic heart disease or dilated cardiomyopathy undergoing heart transplantation. Circulation.

[51] Van Den Heuvel A.F.M., Van Veldhuisen D.J., Van Der Wall E.E., Blanksma P.K., Siebelink H.M.J., Vaalburg W.M., Van Gilst W.H., Crijns H.J.G.M. (2000). Regional myocardial blood flow reserve impairment and metabolic changes suggesting myocardial ischemia in patients with idiopathic dilated cardiomyopathy. J. Am. Coll. Cardiol..

[52] Eisner D.A., Caldwell J.L., Kistamás K., Trafford A.W. (2017). Calcium and Excitation-Contraction Coupling in the Heart. Circ. Res..

[53] Brittsan A.G., Kranias E.G. (2000). Phospholamban and cardiac contractile function. J. Mol. Cell. Cardiol..

[54] Kranias E.G., Hajjar R.J. (2012). Modulation of cardiac contractility by the phopholamban/SERCA2a regulatome. Circ. Res..

[55] MacLennan D.H., Abu-Abed M., Kang C.H. (2002). Structure-function relationships in Ca^2+^ cycling proteins. J. Mol. Cell. Cardiol..

[56] MacLennan D.H., Kranias E.G. (2003). Phospholamban: A crucial regulator of cardiac contractility. Nat. Rev. Mol. Cell Biol..

[57] Limas C.J., Olivari M.T., Goldenberg I.F., Levine T.B., Benditt D.G., Simon A. (1987). Calcium uptake by cardiac sarcoplasmic reticulum in human dilated cardiomyopathy. Cardiovasc. Res..

[58] Bellinger A.M., Reiken S., Carlson C., Mongillo M., Liu X., Rothman L., Matecki S., Lacampagne A., Marks A.R. (2009). Hypernitrosylated ryanodine receptor calcium release channels are leaky in dystrophic muscle. Nat. Med..

[59] Hasenfuss G., Reinecke H., Studer R., Meyer M., Pieske B., Holtz J., Holubarsch C., Posival H., Just H., Drexler H. (1994). Relation between myocardial function and expression of sarcoplasmic reticulum Ca^2+^-ATPase in failing and nonfailing human myocardium. Circ. Res..

[60] Dash R., Frank K.F., Carr A.N., Moravec C.S., Kranias E.G. (2001). Gender influences on sarcoplasmic reticulum Ca^2+^-handling in failing human myocardium. J. Mol. Cell. Cardiol..

[61] Meyer M., Schillinger W., Pieske B., Holubarsch C., Heilmann C., Posival H., Kuwajima G., Mikoshiba K., Just H., Hasenfuss G. (1995). Alterations of sarcoplasmic reticulum proteins in failing human dilated cardiomyopathy. Circulation.

[62] Townsend D.W., Yasuda S., Metzger J. (2007). Cardiomyopathy of Duchenne muscular dystrophy: Pathogenesis and prospect of membrane sealants as a new therapeutic approach. Expert Rev. Cardiovasc. Ther..

[63] Houang E.M., Sham Y.Y., Bates F.S., Metzger J.M. (2018). Muscle membrane integrity in Duchenne muscular dystrophy: Recent advances in copolymer-based muscle membrane stabilizers. Skelet. Muscle.

[64] Claflin D.R., Brooks S.V. (2008). Direct observation of failing fibers in muscles of dystrophic mice provides mechanistic insight into muscular dystrophy. Am. J. Physiol. Cell Physiol..

[65] Allen D.G., Gervasio O.L., Yeung E.W., Whitehead N.P. (2010). Calcium and the damage pathways in muscular dystrophy. Can. J. Physiol. Pharmacol..

[66] Prosser B.L., Khairallah R.J., Ziman A.P., Ward C.W., Lederer W.J. (2013). X-ROS signaling in the heart and skeletal muscle: Stretch-dependent local ROS regulates [Ca^2+^]i. J. Mol. Cell. Cardiol..

[67] Danialou G., Comtois A.S., Dudley R., Karpati G., Vincent G., Des Rosiers C., Petrof B.J. (2001). Dystrophin-deficient cardiomyocytes are abnormally vulnerable to mechanical stress-induced contractile failure and injury. FASEB J..

[68] Townsend D., Turner I., Yasuda S., Martindale J., Davis J., Shillingford M., Kornegay J.N., Metzger J.M. (2010). Chronic administration of membrane sealant prevents severe cardiac injury and ventricular dilatation in dystrophic dogs. J. Clin. Investig..

[69] Prosser B.L., Ward C.W., Jonathan Lederer W. (2013). X-ROS signalling is enhanced and graded by cyclic cardiomyocyte stretch. Cardiovasc. Res..

[70] Williams I.A., Allen D.G. (2007). The role of reactive oxygen species in the hearts of dystrophin-deficient mdx mice. Am. J. Physiol. Heart Circ. Physiol..

[71] Ullrich N.D., Fanchaouy M., Gusev K., Shirokova N., Niggli E. (2009). Hypersensitivity of excitation-contraction coupling in dystrophic cardiomyocytes. Am. J. Physiol. Heart Circ. Physiol..

[72] Prosser B.L., Ward C.W., Lederer W.J. (2011). X-ROS signaling: Rapid mechano-chemo transduction in heart. Science.

[73] Prins K.W., Asp M.L., Zhang H., Wang W., Metzger J.M. (2016). Microtubule-Mediated Misregulation of Junctophilin-2 Underlies T-Tubule Disruptions and Calcium Mishandling in mdx Mice. JACC Basic Transl. Sci..

[74] Koenig X., Rubi L., Obermair G.J., Cervenka R., Dang X.B., Lukacs P., Kummer S., Bittner R.E., Kubista H., Todt H. (2014). Enhanced currents through L-type calcium channels in cardiomyocytes disturb the electrophysiology of the dystrophic heart. Am. J. Physiol. Heart Circ. Physiol..

[75] Valladares D., Almarza G., Contreras A., Pavez M., Buvinic S., Jaimovich E., Casas M. (2013). Electrical stimuli are anti-apoptotic in skeletal muscle via extracellular ATP. Alteration of this signal in Mdx mice is a likely cause of dystrophy. PLoS ONE.

[76] Seidlmayer L.K., Kuhn J., Berbner A., Arias-Loza P.A., Williams T., Kaspar M., Czolbe M., Kwong J.Q., Molkentin J.D., Heinze K.G. (2016). Inositol 1,4,5-Trisphosphate-mediated sarcoplasmic reticulum-mitochondrial crosstalk influences adenosine triphosphate production via mitochondrial Ca^2+^ uptake through the mitochondrial ryanodine receptor in cardiac myocytes. Cardiovasc. Res..

[77] Chen X., Zhang X., Kubo H., Harris D.M., Mills G.D., Moyer J., Berretta R., Potts S.T., Marsh J.D., Houser S.R. (2005). Ca^2+^ influx-induced sarcoplasmic reticulum Ca^2+^ overload causes mitochondrial-dependent apoptosis in ventricular myocytes. Circ. Res..

[78] Luongo T.S., Lambert J.P., Gross P., Nwokedi M., Lombardi A.A., Shanmughapriya S., Carpenter A.C., Kolmetzky D., Gao E., Van Berlo J.H. (2017). The mitochondrial Na^+^/Ca^2+^ exchanger is essential for Ca^2+^ homeostasis and viability. Nature.

[79] Jung C., Martins A.S., Niggli E., Shirokova N. (2008). Dystrophic cardiomyopathy: Amplification of cellular damage by Ca^2+^ signalling and reactive oxygen species-generating pathways. Cardiovasc. Res..

[80] Kyrychenko V., Poláková E., Janíček R., Shirokova N. (2015). Mitochondrial dysfunctions during progression of dystrophic cardiomyopathy. Cell Calcium.

[81] Viola H.M., Adams A.M., Davies S.M.K., Fletcher S., Filipovska A., Hool L.C. (2014). Impaired functional communication between the L-type calcium channel and mitochondria contributes to metabolic inhibition in the mdx heart. Proc. Natl. Acad. Sci. USA.

[82] Millay D.P., Sargent M.A., Osinska H., Baines C.P., Barton E.R., Vuagniaux G., Sweeney H.L., Robbins J., Molkentin J.D. (2008). Genetic and pharmacologic inhibition of mitochondrial-dependent necrosis attenuates muscular dystrophy. Nat. Med..

[83] Giorgio V., Soriano M.E., Basso E., Bisetto E., Lippe G., Forte M.A., Bernardi P. (2010). Cyclophilin D in mitochondrial pathophysiology. Biochim. Biophys. Acta Bioenerg..

[84] Finsterer J., Stöllberger C. (2003). The heart in human dystrophinopathies. Cardiology.

[85] Connuck D.M., Sleeper L.A., Colan S.D., Cox G.F., Towbin J.A., Lowe A.M., Wilkinson J.D., Orav E.J., Cuniberti L., Salbert B.A. (2008). Characteristics and outcomes of cardiomyopathy in children with Duchenne or Becker muscular dystrophy: A comparative study from the Pediatric Cardiomyopathy Registry. Am. Heart J..

[86] Cox G.F., Kunkel L.M. (1997). Dystrophies and heart disease. Curr. Opin. Cardiol..

[87] Frankel K.A., Rosser R.J. (1976). The pathology of the heart in progressive muscular dystrophy: Epimyocardial fibrosis. Hum. Pathol..

[88] Giglio V., Pasceri V., Messano L., Mangiola F., Pasquini L., Dello Russo A., Damiani A., Mirabella M., Galluzzi G., Tonali P. (2003). Ultrasound tissue characterization detects preclinical myocardial structural changes in children affected by Duchenne muscular dystrophy. J. Am. Coll. Cardiol..

[89] Sasaki K., Sakata K., Kachi E., Hirata S., Ishihara T., Ishikawa K. (1998). Sequential changes in cardiac structure and function in patients with Duchenne type muscular dystrophy: A two-dimensional echocardiographic study. Am. Heart J..

[90] Takenaka A., Yokota M., Iwase M., Miyaguchi K., Hayashi H., Saito H. (1993). Discrepancy between systolic and diastolic dysfunction of the left ventricle in patients with Duchenne muscular dystrophy. Eur. Heart J..

[91] Ikeda Y., Ross J. (2000). Models of dilated cardiomyopathy in the mouse and the hamster. Curr. Opin. Cardiol..

[92] Arber S., Hunter J.J., Ross J., Hongo M., Sansig G., Borg J., Perriard J.C., Chien K.R., Caroni P. (1997). MLP-deficient mice exhibit a disruption of cardiac cytoarchitectural organization, dilated cardiomyopathy, and heart failure. Cell.

[93] Heckmann M.B., Bauer R., Jungmann A., Winter L., Rapti K., Strucksberg K.H., Clemen C.S., Li Z., Schröder R., Katus H.A. (2016). AAV9-mediated gene transfer of desmin ameliorates cardiomyopathy in desmin-deficient mice. Gene Ther..

[94] Dai S., Yuan F., Mu J., Li C., Chen N., Guo S., Kingery J., Prabhu S.D., Bolli R., Rokosh G. (2010). Chronic AMD3100 antagonism of SDF-1α-CXCR4 exacerbates cardiac dysfunction and remodeling after myocardial infarction. J. Mol. Cell. Cardiol..

[95] Michael L.H., Entman M.L., Hartley C.J., Youker K.A., Zhu J., Hall S.R., Hawkins H.K., Berens K., Ballantyne C.M. (1995). Myocardial ischemia and reperfusion: A murine model. Am. J. Physiol. Heart Circ. Physiol..

[96] Weinstein D.M., Mihm M.J., Bauer J.A. (2000). Cardiac peroxynitrite formation and left ventricular dysfunction following doxorubicin treatment in mice. J. Pharmacol. Exp. Ther..

[97] Robert J. (2007). Long-term and short-term models for studying anthracycline cardiotoxicity and protectors. Cardiovasc. Toxicol..

[98] Teerlink J.R., Pfeffer J.M., Pfeffer M.A. (1994). Progressive ventricular remodeling in response to diffuse isoproterenol-induced myocardial necrosis in rats. Circ. Res..

[99] Oudit G.Y., Crackower M.A., Eriksson U., Sarao R., Kozieradzki I., Sasaki T., Irie-Sasaki J., Gidrewicz D., Rybin V.O., Wada T. (2003). Phosphoinositide 3-Kinase γ-Deficient Mice Are Protected From Isoproterenol-Induced Heart Failure. Circulation.

[100] Hainsey T.A., Senapati S., Kuhn D.E., Rafael J.A. (2003). Cardiomyopathic features associated with muscular dystrophy are independent of dystrophin absence in cardiovasculature. Neuromuscul. Disord..

[101] Janssen P.M.L., Hiranandani N., Mays T.A., Rafael-Fortney J.A. (2005). Utrophin deficiency worsens cardiac contractile dysfunction present in dystrophin-deficient mdx mice. Am. J. Physiol. Heart Circ. Physiol..

[102] Grady R.M., Teng H., Nichol M.C., Cunningham J.C., Wilkinson R.S., Sanest J.R. (1997). Skeletal and cardiac myopathies in mice lacking utrophin and dystrophin: A model for Duchenne muscular dystrophy. Cell.

[103] Deconinck A.E., Rafael J.A., Skinner J.A., Brown S.C., Potter A.C., Metzinger L., Watt D.J., Dickson J.G., Tinsley J.M., Davies K.E. (1997). Utrophin-dystrophin-deficient mice as a model for Duchenne muscular dystrophy. Cell.

[104] Hearse D.J., Sutherland F.J. (2000). Experimental Models for the Study of Cardiovascular Function and Disease Defining the Question and Identifying the Model. Pharmacol. Res..

[105] Cui J., Li J., Mathison M., Tondato F., Mulkey S.P., Micko C., Chronos N.A.F., Robinson K.A. (2005). A clinically relevant large-animal model for evaluation of tissue-engineered cardiac surgical patch materials. Cardiovasc. Revasc. Med..

[106] Jugdutt B.I., Jelani A., Palaniyappan A., Idikio H., Uweira R.E., Menon V., Jugdutt C.E. (2010). Aging-related early changes in markers of ventricular and matrix remodeling after reperfused st-segment elevation myocardial infarction in the canine model: Effect of early therapy with an angiotensin II type 1 receptor blocker. Circulation.

[107] Lee S.T., White A.J., Matsushita S., Malliaras K., Steenbergen C., Zhang Y., Li T.S., Terrovitis J., Yee K., Simsir S. (2011). Intramyocardial injection of autologous cardiospheres or cardiosphere-derived cells preserves function and minimizes adverse ventricular remodeling in pigs with heart failure post-myocardial infarction. J. Am. Coll. Cardiol..

[108] Gorman J.H., Gorman R.C., Plappert T., Jackson B.M., Hiramatsu Y., St. John-Sutton M.G., Edmunds J. (1998). Infarct size and location determine development of mitral regurgitation in the sheep model. J. Thorac. Cardiovasc. Surg..

[109] Sampaolesi M., Blot S., D’Antona G., Granger N., Tonlorenzi R., Innocenzi A., Mognol P., Thibaud J.L., Galvez B.G., Barthélémy I. (2006). Mesoangioblast stem cells ameliorate muscle function in dystrophic dogs. Nature.

[110] Wang Z., Kuhr C.S., Allen J.M., Blankinship M., Gregorevic P., Chamberlain J.S., Tapscott S.J., Storb R. (2007). Sustained AAV-mediated dystrophin expression in a canine model of duchenne muscular dystrophy with a brief course of immunosuppression. Mol. Ther..

[111] Sun N., Yazawa M., Liu J., Han L., Sanchez-Freire V., Abilez O.J., Navarrete E.G., Hu S., Wang L., Lee A. (2012). Patient-specific induced pluripotent stem cells as a model for familial dilated cardiomyopathy. Sci. Transl. Med..

[112] Siu C.W., Lee Y.K., Ho J.C.Y., Lai W.H., Chan Y.C., Ng K.M., Wong L.Y., Au K.W., Lau Y.M., Zhang J. (2012). Modeling of lamin A/C mutation premature cardiac aging using patient-specific induced pluripotent stem cells. Aging Albany NY.

[113] Tse H.F., Ho J.C.Y., Choi S.W., Lee Y.K., Butler A.W., Ng K.M., Siu C.W., Simpson M.A., Lai W.H., Chan Y.C. (2013). Patient-specific induced-pluripotent stem cells-derived cardiomyocytes recapitulate the pathogenic phenotypes of dilated cardiomyopathy due to a novel DES mutation identified by whole exome sequencing. Hum. Mol. Genet..

[114] Lin B., Li Y., Han L., Kaplan A.D., Ao Y., Kalra S., Bett G.C.L., Rasmusson R.L., Denning C., Yang L. (2015). Modeling and study of the mechanism of dilated cardiomyopathy using induced pluripotent stem cells derived from individuals with duchenne muscular dystrophy. DMM Dis. Model. Mech..

[115] Recchia F.A., Lionetti V. (2007). Animal models of dilated cardiomyopathy for translational research. Vet. Res. Commun..

[116] Chu G., Haghighi K., Kranias E.G. (2002). From mouse to man: Understanding heart failure through genetically altered mouse models. J. Cardiac Fail..

[117] Wang Q.D., Bohlooly M., Sjöquist P.O. (2004). Murine models for the study of congestive heart failure: Implications for understanding molecular mechanisms and for drug discovery. J. Pharmacol. Toxicol. Methods.

[118] Grady R.M., Grange R.W., Lau K.S., Maimone M.M., Nichol M.C., Stull J.T., Sanes J.R. (1999). Role for α-dystrobrevin in the pathogenesis of dystrophin-dependent muscular dystrophies. Nat. Cell Biol..

[119] Li D., Long C., Yue Y., Duan D. (2009). Sub-physiological sarcoglycan expression contributes to compensatory muscle protection in mdx mice. Hum. Mol. Genet..

[120] Han R., Rader E.P., Levy J.R., Bansal D., Campbell K.P. (2011). Dystrophin deficiency exacerbates skeletal muscle pathology in dysferlin-null mice. Skelet. Muscle.

[121] Hosur V., Kavirayani A., Riefler J., Carney L.M.B., Lyons B., Gott B., Cox G.A., Shultz L.D. (2012). Dystrophin and dysferlin double mutant mice: A novel model for rhabdomyosarcoma. Cancer Genet..

[122] Banks G.B., Combs A.C., Odom G.L., Bloch R.J., Chamberlain J.S. (2014). Muscle Structure Influences Utrophin Expression in mdx Mice. PLoS Genet..

[123] Gawlik K.I., Holmberg J., Durbeej M. (2014). Loss of dystrophin and β-sarcoglycan significantly exacerbates the phenotype of laminin α2 chain-deficient animals. Am. J. Pathol..

[124] Monnet E., Chachques J.C. (2005). Animal models of heart failure: What is new?. Ann. Thorac. Surg..

[125] Dixon J.A., Spinale F.G. (2009). Large animal models of heart failure; A critical link in the translation of basic science to clinical practice. Circ. Heart Fail..

[126] Camacho P., Fan H., Liu Z., He J.Q. (2016). Small mammalian animal models of heart disease. Am. J. Cardiovasc. Dis..

[127] Valentine B.A., Cummings J.F., Cooper B.J. (1989). Development of Duchenne-type cardiomyopathy. Morphologic studies in a canine model. Am. J. Pathol..

[128] Moise N.S., Valentine B.A., Brown C.A., Erb H.N., Beck K.A., Cooper B.J., Gilmour R.F. (1991). Duchenne’s cardiomyopathy in a canine model: Electrocardiographic and echocardiographic studies. J. Am. Coll. Cardiol..

[129] Devaux J.Y., Cabane L., Esler M., Flaouters H., Duboc D. (1993). Non-invasive evaluation of the cardiac function in Golden Retriever dogs by radionuclide angiography. Neuromuscul. Disord..

[130] Takahashi K., Tanabe K., Ohnuki M., Narita M., Ichisaka T., Tomoda K., Yamanaka S. (2007). Induction of Pluripotent Stem Cells from Adult Human Fibroblasts by Defined Factors. Cell.

[131] Guan X., Mack D.L., Moreno C.M., Strande J.L., Mathieu J., Shi Y., Markert C.D., Wang Z., Liu G., Lawlor M.W. (2014). Dystrophin-deficient cardiomyocytes derived from human urine: New biologic reagents for drug discovery. Stem Cell Res..

[132] Tsurumi F., Baba S., Yoshinaga D., Umeda K., Hirata T., Takita J., Heike T. (2019). The intracellular Ca^2+^ concentration is elevated in cardiomyocytes differentiated from hiPSCs derived from a Duchenne muscular dystrophy patient. PLoS ONE.

[133] Verhaart I.E.C., Aartsma-Rus A. (2019). Therapeutic developments for Duchenne muscular dystrophy. Nat. Rev. Neurol..

[134] Griggs R.C., Moxley R.T., Mendell J.R., Fenichel G.M., Brooke M.H., Pestronk A., Miller J.P. (1991). Prednisone in Duchenne Dystrophy: A Randomized, Controlled Trial Defining the Time Course and Dose Response. Arch. Neurol..

[135] Biggar W.D., Harris V.A., Eliasoph L., Alman B. (2006). Long-term benefits of deflazacort treatment for boys with Duchenne muscular dystrophy in their second decade. Neuromuscul. Disord..

[136] Spurney C.F. (2011). Cardiomyopathy of duchenne muscular dystrophy: Current understanding and future directions. Muscle Nerve.

[137] Schram G., Fournier A., Leduc H., Dahdah N., Therien J., Vanasse M., Khairy P. (2013). All-cause mortality and cardiovascular outcomes with prophylactic steroid therapy in Duchenne muscular dystrophy. J. Am. Coll. Cardiol..

[138] Raman S.V., Cripe L.H. (2015). Glucocorticoid therapy for Duchenne cardiomyopathy: A Hobson’s choice?. J. Am. Heart Assoc..

[139] Barber B.J., Andrews J.G., Lu Z., West N.A., Meaney F.J., Price E.T., Gray A., Sheehan D.W., Pandya S., Yang M. (2013). Oral corticosteroids and onset of cardiomyopathy in Duchenne muscular dystrophy. J. Pediatr..

[140] Griggs R.C., Herr B.E., Reha A., Elfring G., Atkinson L., Cwik V., Mccoll E., Tawil R., Pandya S., Mcdermott M.P. (2013). Corticosteroids in Duchenne muscular dystrophy: Major variations in practice. Muscle Nerve.

[141] Hoffman E.P., Reeves E., Damsker J., Nagaraju K., McCall J.M., Connor E.M., Bushby K. (2012). Novel Approaches to Corticosteroid Treatment in Duchenne Muscular Dystrophy. Phys. Med. Rehabil. Clin. N. Am..

[142] Bauer R., Straub V., Blain A., Bushby K., MacGowan G.A. (2009). Contrasting effects of steroids and angiotensin-converting-enzyme inhibitors in a mouse model of dystrophin-deficient cardiomyopathy. Eur. J. Heart Fail..

[143] Janssen P.M.L., Murray J.D., Schill K.E., Rastogi N., Schultz E.J., Tran T., Raman S.V., Rafael-Fortney J.A. (2014). Prednisolone attenuates improvement of cardiac and skeletal contractile function and histopathology by lisinopril and spironolactone in the mdx mouse model of duchenne muscular dystrophy. PLoS ONE.

[144] Ogata H., Ishikawa Y., Ishikawa Y., Minami R. (2009). Beneficial effects of beta-blockers and angiotensin-converting enzyme inhibitors in Duchenne muscular dystrophy. J. Cardiol..

[145] Dikalov S.I., Nazarewicz R.R. (2013). Angiotensin II-induced production of mitochondrial reactive oxygen species: Potential mechanisms and relevance for cardiovascular disease. Antioxid. Redox Signal..

[146] Dasgupta C., Zhang L. (2011). Angiotensin II receptors and drug discovery in cardiovascular disease. Drug Discov. Today.

[147] Kawai T., Forrester S.J., O’Brian S., Baggett A., Rizzo V., Eguchi S. (2017). AT1 receptor signaling pathways in the cardiovascular system. Pharmacol. Res..

[148] Bernal J., Pitta S.R., Thatai D. (2006). Role of the renin-angiotensin-aldosterone system in diastolic heart failure: Potential for pharmacologic intervention. Am. J. Cardiovasc. Drugs.

[149] Duboc D., Meune C., Pierre B., Wahbi K., Eymard B., Toutain A., Berard C., Vaksmann G., Weber S., Bécane H.M. (2007). Perindopril preventive treatment on mortality in Duchenne muscular dystrophy: 10 years’ follow-up. Am. Heart J..

[150] Bangalore S., Fakheri R., Toklu B., Ogedegbe G., Weintraub H., Messerli F.H. (2016). Angiotensin-Converting Enzyme Inhibitors or Angiotensin Receptor Blockers in Patients Without Heart Failure? Insights from 254,301 Patients from Randomized Trials. Mayo Clin. Proc..

[151] Triposkiadis F., Karayannis G., Giamouzis G., Skoularigis J., Louridas G., Butler J. (2009). The Sympathetic Nervous System in Heart Failure. Physiology, Pathophysiology, and Clinical Implications. J. Am. Coll. Cardiol..

[152] Klapholz M. (2009). Β-Blocker Use for the Stages of Heart Failure. Mayo Clin. Proc..

[153] Jefferies J.L., Eidem B.W., Belmont J.W., Craigen W.J., Ware S.M., Fernbach S.D., Neish S.R., Smith E.O.B., Towbin J.A. (2005). Genetic predictors and remodeling of dilated cardiomyopathy in muscular dystrophy. Circulation.

[154] Kajimoto H., Ishigaki K., Okumura K., Tomimatsu H., Nakazawa M., Saito K., Osawa M., Nakanishi T. (2006). Beta-blocker therapy for cardiac dysfunction in patients with muscular dystrophy. Circ. J..

[155] Matsumura T., Tamura T., Kuru S., Kikuchi Y., Kawai M. (2010). Carvedilol can prevent cardiac events in duchenne muscular dystrophy. Intern. Med..

[156] Viollet L., Thrush P.T., Flanigan K.M., Mendell J.R., Allen H.D. (2012). Effects of angiotensin-converting enzyme inhibitors and/or beta blockers on the cardiomyopathy in duchenne muscular dystrophy. Am. J. Cardiol..

[157] Raman S.V., Hor K.N., Mazur W., Halnon N.J., Kissel J.T., He X., Tran T., Smart S., McCarthy B., Taylor M.D. (2015). Eplerenone for early cardiomyopathy in Duchenne muscular dystrophy: A randomised, double-blind, placebo-controlled trial. Lancet Neurol..

[158] Spurney C.F., Guerron A.D., Yu Q., Sali A., van der Meulen J.H., Hoffman E.P., Nagaraju K. (2011). Membrane Sealant Poloxamer P188 Protects Against Isoproterenol Induced Cardiomyopathy in Dystrophin Deficient Mice. BMC Cardiovasc. Disord..

[159] Martindale J.J., Metzger J.M. (2014). Uncoupling of increased cellular oxidative stress and myocardial ischemia reperfusion injury by directed sarcolemma stabilization. J. Mol. Cell. Cardiol..

[160] Ward M.L., Williams I.A., Chu Y., Cooper P.J., Ju Y.K., Allen D.G. (2008). Stretch-activated channels in the heart: Contributions to length-dependence and to cardiomyopathy. Prog. Biophys. Mol. Biol..

[161] Fauconnier J., Thireau J., Reiken S., Cassan C., Richard S., Matecki S., Marks A.R., Lacampagne A. (2010). Leaky RyR2 trigger ventricular arrhythmias in Duchenne muscular dystrophy. Proc. Natl. Acad. Sci. USA.

[162] Shin J.H., Bostick B., Yue Y., Hajjar R., Duan D. (2011). SERCA2a gene transfer improves electrocardiographic performance in aged mdx mice. J. Transl. Med..

[163] Hoshijima M., Ikeda Y., Iwanaga Y., Minamisawa S., Date M.O., Gu Y., Iwatate M., Li M., Wang L., Wilson J.M. (2002). Chronic suppression of heart-failure progression by a pseudophosphorylated mutant of phospholamban via in vivo cardiac rAAV gene delivery. Nat. Med..

[164] Dieterle T., Meyer M., Gu Y., Belke D.D., Swanson E., Iwatate M., Hollander J., Peterson K.L., Ross J., Dillmann W.H. (2005). Gene transfer of a phospholamban-targeted antibody improves calcium handling and cardiac function in heart failure. Cardiovasc. Res..

[165] Yamada M., Ikeda Y., Yano M., Yoshimura K., Nishino S., Aoyama H., Wang L., Aoki H., Matsuzaki M. (2006). Inhibition of protein phosphatase 1 by inhibitor-2 gene delivery ameliorates heart failure progression in genetic cardiomyopathy. FASEB J..

[166] Houang E.M., Bartos J., Hackel B.J., Lodge T.P., Yannopoulos D., Bates F.S., Metzger J.M. (2019). Cardiac Muscle Membrane Stabilization in Myocardial Reperfusion Injury. JACC Basic Transl. Sci..

[167] Weisleder N., Takizawa N., Lin P., Wang X., Cao C., Zhang Y., Tan T., Ferrante C., Zhu H., Chen P.J. (2012). Recombinant MG53 protein modulates therapeutic cell membrane repair in treatment of muscular dystrophy. Sci. Transl. Med..

[168] Vandebrouck C., Martin D., Van Schoor M.C., Debaix H., Gailly P. (2002). Involvement of TRPC in the abnormal calcium influx observed in dystrophic (mdx) mouse skeletal muscle fibers. J. Cell Biol..

[169] Huang F., Shan J., Reiken S., Wehrens X.H.T., Marks A.R. (2006). Analysis of calstabin2 (FKBP12.6)-ryanodine receptor interactions: Rescue of heart failure by calstabin2 in mice. Proc. Natl. Acad. Sci. USA.

[170] Capogrosso R.F., Mantuano P., Uaesoontrachoon K., Cozzoli A., Giustino A., Dow T., Srinivassane S., Filipovic M., Bell C., Vandermeulen J. (2018). Ryanodine channel complex stabilizer compound S48168/ARM210 as a disease modifier in dystrophin-deficient mdx mice: Proof-of-concept study and independent validation of efficacy. FASEB J..

[171] Del Monte F., Harding S.E., Schmidt U., Matsui T., Kang Z.B., Dec G.W., Gwathmey J.K., Rosenzweig A., Hajjar R.J. (1999). Restoration of contractile function in isolated cardiomyocytes from failing human hearts by gene transfer of SERCA2a. Circulation.

[172] Miyamoto M.I., Del Monte F., Schmidt U., DiSalvo T.S., Kang Z.B., Matsui T., Guerrero J.L., Gwathmey J.K., Rosenzweig A., Hajjar R.J. (2000). Adenoviral gene transfer of SERCA2A improves left-ventricular function in aortic-banded rats in transition to heart failure. Proc. Natl. Acad. Sci. USA.

[173] Del Monte F., Williams E., Lebeche D., Schmidt U., Rosenzweig A., Gwathmey J.K., Lewandowski E.D., Hajjar R.J. (2001). Improvement in survival and cardiac metabolism after gene transfer of sarcoplasmic reticulum Ca^2+^-ATPase in a rat model of heart failure. Circulation.

[174] Sakata S., Lebeche D., Sakata N., Sakata Y., Chemaly E.R., Liang L.F., Tsuji T., Takewa Y., del Monte F., Peluso R. (2007). Restoration of mechanical and energetic function in failing aortic-banded rat hearts by gene transfer of calcium cycling proteins. J. Mol. Cell. Cardiol..

[175] Niwano K., Arai M., Koitabashi N., Watanabe A., Ikeda Y., Miyoshi H., Kurabayashi M. (2008). Lentiviral vector-mediated SERCA2 gene transfer protects against heart failure and left ventricular remodeling after myocardial Infarction in rats. Mol. Ther..

[176] Byrne M.J., Power J.M., Preovolos A., Mariani J.A., Hajjar R.J., Kaye D.M. (2008). Recirculating cardiac delivery of AAV2/1SERCA2a improves myocardial function in an experimental model of heart failure in large animals. Gene Ther..

[177] Mariani J.A., Smolic A., Preovolos A., Byrne M.J., Power J.M., Kaye D.M. (2011). Augmentation of left ventricular mechanics by recirculation-mediated AAV2/1SERCA2a gene delivery in experimental heart failure. Eur. J. Heart Fail..

[178] Jessup M., Greenberg B., Mancini D., Cappola T., Pauly D.F., Jaski B., Yaroshinsky A., Zsebo K.M., Dittrich H., Hajjar R.J. (2011). Calcium upregulation by percutaneous administration of gene therapy in cardiac disease (CUPID): A phase 2 trial of intracoronary gene therapy of sarcoplasmic reticulum Ca^2+^-ATPase in patients with advanced heart failure. Circulation.

[179] Zsebo K., Yaroshinsky A., Rudy J.J., Wagner K., Greenberg B., Jessup M., Hajjar R.J. (2014). Long-term effects of AAV1/SERCA2a gene transfer in patients with severe heart failure: Analysis of recurrent cardiovascular events and mortality. Circ. Res..

[180] Greenberg B., Butler J., Felker G.M., Ponikowski P., Voors A.A., Desai A.S., Barnard D., Bouchard A., Jaski B., Lyon A.R. (2016). Calcium upregulation by percutaneous administration of gene therapy in patients with cardiac disease (CUPID 2): A randomised, multinational, double-blind, placebo-controlled, phase 2b trial. Lancet.

[181] Kadambi V.J., Ponniah S., Harrer J.M., Hoit B.D., Dorn G.W., Walsh R.A., Kranias E.G. (1996). Cardiac-specific overexpression of phospholamban alters calcium kinetics and resultant cardiomyocyte mechanics in transgenic mice. J. Clin. Investig..

[182] Luo W., Grupp I.L., Harrer J., Ponniah S., Grupp G., Duffy J.J., Doetschman T., Kranias E.G. (1994). Targeted ablation of the phospholamban gene is associated with markedly enhanced myocardial contractility and loss of β-agonist stimulation. Circ. Res..

[183] Luo W., Wolska B.M., Grupp I.L., Harrer J.M., Haghighi K., Ferguson D.G., Slack J.P., Grupp G., Doetschman T., Solaro R.J. (1996). Phospholamban gene dosage effects in the mammalian heart. Circ. Res..

[184] Minamisawa S., Hoshijima M., Chu G., Ward C.A., Frank K., Gu Y., Martone M.E., Wang Y., Ross J., Kranias E.G. (1999). Chronic phospholamban-sarcoplasmic reticulum calcium atpase interaction is the critical calcium cycling defect in dilated cardiomyopathy. Cell.

[185] Iwanaga Y., Hoshijima M., Gu Y., Iwatate M., Dieterle T., Ikeda Y., Date M.O., Chrast J., Matsuzaki M., Peterson K.L. (2004). Chronic phospholamban inhibition prevents progressive cardiac dysfunction and pathological remodeling after infarction in rats. J. Clin. Investig..

[186] Kaye D.M., Preovolos A., Marshall T., Byrne M., Hoshijima M., Hajjar R., Mariani J.A., Pepe S., Chien K.R., Power J.M. (2007). Percutaneous Cardiac Recirculation-Mediated Gene Transfer of an Inhibitory Phospholamban Peptide Reverses Advanced Heart Failure in Large Animals. J. Am. Coll. Cardiol..

[187] Miyazaki Y., Ikeda Y., Shiraishi K., Fujimoto S.N., Aoyama H., Yoshimura K., Inui M., Hoshijima M., Kasahara H., Aoki H. (2012). Heart failure-inducible gene therapy targeting protein phosphatase 1 prevents progressive left ventricular remodeling. PLoS ONE.

[188] Rohman M.S., Emoto N., Takeshima Y., Yokoyama M., Matsuo M. (2003). Decreased mAKAP, ryanodine receptor, and SERCA2a gene expression in mdx hearts. Biochem. Biophys. Res. Commun..

[189] Goonasekera S.A., Lam C.K., Millay D.P., Sargent M.A., Hajjar R.J., Kranias E.G., Molkentin J.D. (2011). Mitigation of muscular dystrophy in mice by SERCA overexpression in skeletal muscle. J. Clin. Investig..

[190] Mázala D.A.G., Pratt S.J.P., Chen D., Molkentin J.D., Lovering R.M., Chin E.R. (2015). SERCA1 overexpression minimizes skeletal muscle damage in dystrophic mouse models. Am. J. Physiol. Cell Physiol..

[191] Ikeda Y., Martone M., Gu Y., Hoshijima M., Thor A., Oh S.S., Peterson K.L., Ross J. (2000). Altered membrane proteins and permeability correlate with cardiac dysfunction in cardiomyopathic hamsters. Am. J. Physiol. Heart Circ. Physiol..

[192] Voit A., Patel V., Pachon R., Shah V., Bakhutma M., Kohlbrenner E., McArdle J.J., Dell’Italia L.J., Mendell J.R., Xie L.H. (2017). Reducing sarcolipin expression mitigates Duchenne muscular dystrophy and associated cardiomyopathy in mice. Nat. Commun..

[193] Chu G., Luo W., Slack J.P., Tilgmann C., Sweet W.E., Spindler M., Saupe K.W., Boivin G.P., Moravec C.S., Matlib M.A. (1996). Compensatory mechanisms associated with the hyperdynamic function of phospholamban-deficient mouse hearts. Circ. Res..

[194] Haghighi K., Kolokathis F., Pater L., Lynch R.A., Asahi M., Gramolini A.O., Fan G.C., Tsiapras D., Hahn H.S., Adamopoulos S. (2003). Human phospholamban null results in lethal dilated cardiomyopathy revealing a critical difference between mouse and human. J. Clin. Investig..

[195] Schmitt J.P., Kamisago M., Asahi M., Hua Li G., Ahmad F., Mende U., Kranias E.G., MacLennan D.H., Seidman J.G., Seidman C.E. (2003). Dilated cardiomyopathy and heart failure caused by a mutation in phospholamban. Science.

[196] Ha K.N., Mastersona L.R., Hou Z., Verardi R., Walsh N., Veglia G., Robia S.L. (2011). Lethal Arg9Cys phospholamban mutation hinders Ca^2+^-ATPase regulation and phosphorylation by protein kinase A. Proc. Natl. Acad. Sci. USA.

[197] Abrol N., De Tombe P.P., Robia S.L. (2015). Acute inotropic and lusitropic effects of cardiomyopathic R9C mutation of phospholamban. J. Biol. Chem..

[198] Small K.M., Wagoner L.E., Levin A.M., Kardia S.L.R., Liggett S.B. (2002). Synergistic polymorphisms of β 1- and α 2C-adrenergic receptors and the risk of congestive heart failure. N. Engl. J. Med..

[199] Haghighi K., Gregory K.N., Kranias E.G. (2004). Sarcoplasmic reticulum Ca-ATPase-phospholamban interactions and dilated cardiomyopathy. Biochem. Biophys. Res. Commun..

[200] Permyakov E.A., Uversky V.N. (2007). Parvalbumin.

[201] Hapak R.C., Zhao H., Boschi J.M., Henzl M.T. (1994). Novel avian thymic parvalbumin displays high degree of sequence homology to oncomodulin. J. Biol. Chem..

[202] Pauls T.L., Cox J.A., Berchtold M.W. (1996). The Ca^2+^-binding proteins parvalbumin and oncomodulin and their genes: New structural and functional findings. Biochim. Biophys. Acta Gene Struct. Expr..

[203] Falke J.J., Drake S.K., Hazard A.L., Peersen O.B. (1994). Molecular Tuning of Ion Binding to Calcium Signaling Proteins. Q. Rev. Biophys..

[204] Wahr P.A., Michele D.E., Metzger J.M. (1999). Parvalbumin gene transfer corrects diastolic dysfunction in diseased cardiac myocytes. Proc. Natl. Acad. Sci. USA.

[205] Huq F., Lebeche D., Iyer V., Liao R., Hajjar R.J. (2004). Gene transfer of parvalbumin improves diastolic dysfunction in senescent myocytes. Circulation.

[206] Rodenbaugh D.W., Wang W., Davis J., Edwards T., Potter J.D., Metzger J.M. (2007). Parvalbumin isoforms differentially accelerate cardiac myocyte relaxation kinetics in an animal model of diastolic dysfunction. Am. J. Physiol. Heart Circ. Physiol..

[207] Szatkowski M.L., Westfall M.V., Gomez C.A., Wahr P.A., Michele D.E., DelloRusso C., Turner I.I., Hong K.E., Albayya F.P., Metzger J.M. (2001). In vivo acceleration of heart relaxation performance by parvalbumin gene delivery. J. Clin. Investig..

[208] Michele D.E., Szatkowski M.L., Albayya F.P., Metzger J.M. (2004). Parvalbumin gene delivery improves diastolic function in the aged myocardium in vivo. Mol. Ther..

[209] Schmidt U., Zhu X., Lebeche D., Huq F., Guerrero J.L., Hajjar R.J. (2005). In vivo gene transfer of parvalbumin improves diastolic function in aged rat hearts. Cardiovasc. Res..

[210] Coutu P., Metzger J.M. (2005). Genetic manipulation of calcium-handling proteins in cardiac myocytes. II. Mathematical modeling studies. Am. J. Physiol. Heart Circ. Physiol..

[211] Coutu P., Metzger J.M. (2005). Genetic manipulation of calcium-handling proteins in cardiac myocytes. I. Experimental studies. Am. J. Physiol. Heart Circ. Physiol..

[212] Coutu P., Metzger J.M. (2002). Optimal range for parvalbumin as relaxing agent in adult cardiac myocytes: Gene transfer and mathematical modeling. Biophys. J..

[213] Wang W., Barnabei M.S., Asp M.L., Heinis F.I., Arden E., Davis J., Braunlin E., Li Q., Davis J.P., Potter J.D. (2013). Noncanonical EF-hand motif strategically delays Ca^2+^ buffering to enhance cardiac performance. Nat. Med..

[214] Asp M.L., Sjaastad F.V., Siddiqui J.K., Davis J.P., Metzger J.M. (2016). Effects of Modified Parvalbumin EF-Hand Motifs on Cardiac Myocyte Contractile Function. Biophys. J..

[215] Liao R., Nascimben L., Friedrich J., Gwathmey J.K., Ingwall J.S. (1996). Decreased energy reserve in an animal model of dilated cardiomyopathy: Relationship to contractile performance. Circ. Res..

